# The Impact of Modifiable Risk Factors on the Endothelial Cell Methylome and Cardiovascular Disease Development

**DOI:** 10.31083/FBL26082

**Published:** 2025-01-07

**Authors:** Hashum Sum, Alison C. Brewer

**Affiliations:** 1 School of Cardiovascular and Metabolic Medicine & Sciences, British Heart Foundation Centre of Research Excellence, King’s College London, SE5 9NU London, UK

**Keywords:** modifiable risk factors, cardiovascular disease, endothelial dysfunction, DNA methylation, TETs, DNMTs

## Abstract

Cardiovascular disease (CVD) is the most prevalent cause of mortality and morbidity in the Western world. A common underlying hallmark of CVD is the plaque-associated arterial thickening, termed atherosclerosis. Although the molecular mechanisms underlying the aetiology of atherosclerosis remain unknown, it is clear that both its development and progression are associated with significant changes in the pattern of DNA methylation within the vascular cell wall. The endothelium is the major regulator of vascular homeostasis, and endothelial cell dysfunction (ED) is considered an early marker for atherosclerosis. Thus, it is speculated that changes in DNA methylation within endothelial cells may, in part, be causal in ED, leading to atherosclerosis and CVD generally. This review will evaluate the extensive evidence that environmental risk factors, known to be associated with atherosclerosis, such as diabetes, metabolic disorder, smoking, hypertension and hypercholesterolaemia *etc*. can affect the methylome of the endothelium and consequently act to alter gene transcription and function. Further, the potential mechanisms whereby such risk factors might impact upon the activities and/or specificities of the epigenetic writers and erasers which determine the methylome [the DNA methyl transferases (DNMTs) and Ten Eleven translocases (TETs)] are considered here. Notably, the TET proteins are members of the 2-oxoglutarate-dependent dioxygenase superfamily which require molecular oxygen (O_2_) and *α*-ketoglutarate (*α*-KG) as substrates and iron-2^+^ (Fe II) as a cofactor. This renders their activities subject to modulation by hypoxia, metabolic flux and cellular redox. The potential significance of this, with respect to the impact of modifiable risk factors upon the activities of the TETs and the methylome of the endothelium is discussed.

## 1 Introduction

Cardiovascular disease (CVD) remains the leading cause of death worldwide, accounting for about one-third of all deaths globally [1]. Genome-wide association studies (GWASs) have identified mutations/polymorphisms in genes linked to lipid metabolism, inflammation, vascular function and structural integrity which can influence the risk of developing CVD [2]. However, such genetic predisposition accounts for only a small percentage of overall cases [3]. Rather, the prevalence of CVD is largely due to environmental cues, where modifiable risk factors such as diets high in cholesterol, sodium, and sugar, along with physical inactivity and smoking, are recognised as independent and major contributors to CVD [4]. Notably, the World Health Organization (WHO) has identified air pollution as the most significant risk factor for CVD, contributing to approximately 25% of the global CVD burden [5]. The precise mechanisms which underlie the aetiology and progression of the environmentally-triggered disease remain unknown but typically involve significant changes to the body’s redox balance and inflammation, leading to sustained alterations in biochemical and physiological systems [2,6].

Despite the diverse manifestations of CVD, the pathologies commonly stem from atherosclerosis (AS). AS is a slow, progressive disease of the arterial blood vessels, and is typically associated with chronic low-level inflammation, oxidative stress, and vascular wall remodeling [7]. One of the earliest stages in this remodelling process involves dysfunction of the endothelium, which is a crucial regulator of vascular homeostasis and is therefore a key target for early detection and intervention in CVD [8]. Endothelial dysfunction (ED) is a systemic disorder which is an independent predictor of pathological cardiovascular events, in which the endothelium manifests as more vasoconstrictive, pro-inflammatory and prothrombotic. Evidence suggests that multiple factors, including traditional modifiable cardiovascular risk factors, together with ageing, gender, and emerging risk determinants such as sleep disorders, gut microbiome alterations, clonal haematopoiesis and foetal factors can all act to promote this more dysfunctional endothelial phenotype [9]. The hallmark of ED is a significant reduction in nitric oxide (NO) bioavailability owing to impaired activation of endothelial nitric oxide synthase (eNOS) and/or increased levels of reactive oxygen species (ROS) [10], and this associates clinically with impaired brachial artery flow-mediated dilation [11]. Elucidating the molecular mechanisms which might underlie the development of endothelial dysfunction (ED) such as in response to exposure to environmental CVD risk factors may therefore prove to be critical in the development of effective therapeutic strategies to prevent and/or treat AS [12].

### 1.1 The Role of the Endothelium

The maintenance of vascular integrity is key to the normal functioning of blood vessels and consequently, vascular homeostasis. The vasculature act as the bridge between the blood and the tissues, providing the all-important oxygen and nutrients to ensure cell survival whilst removing any waste and other harmful products for disposal. The general architecture and cellular composition of blood vessels is similar throughout the vascular system. It comprises the tunica externa (outer layer), the tunica media (middle layer), and the intima (inner layer) [13]. However, certain vessel characteristics will predominate according to the different functional requirements at different locations, i.e., arteries, veins, arterioles, venules, and the capillary vessels.

The endothelium—comprising of a single layer of endothelial cells—forms the inner lining of blood vessels, at the intima. Once considered only as a mechanical barrier, we now understand the endothelium to be much more than that. Optimally positioned between the underlying tissue and the blood itself, this semi-permeable barrier is now regarded as a highly dynamic organ [13] which plays critical roles in vascular biology and pathophysiology. It not only helps preserve the structural integrity of blood vessels but also responds to both chemical and physical stimuli via the production of a wide variety of factors.

There are two broad categories that blood vessels fall under—microvasculature and macrovasculature. The microvasculature consists of small arterioles, capillaries, and venules, which play a crucial role in regulating local blood perfusion and facilitating metabolic exchanges between the circulatory system and peripheral tissues. Meanwhile, the macrovasculature are the arteries and veins which are responsible for transporting blood to and from organs [14]. In larger vessels, the intima will consist of not only endothelial cells but also some smooth muscle cells and elastic fibres, which help maintain the vascular tone. However, in the smaller vessels such as the capillaries, the endothelial cells will be the only component of the entire vessel wall [15]. Dysfunction of both micro- and macro-vascular endothelial cells have been shown to be predictors of future cardiovascular events [16–18]. However, in the case of AS, it is dysfunction of the macrovascular endothelial cells of the large (proximal) arteries that is of most clinical relevance [19].

In normal vascular homeostasis, the endothelium also has the role of controlling the transfer of molecules across the vessel wall (in a size-selective manner) via the paracellular pathway (involving interendothelial junctions) or the transcellular pathway (across the endothelial cell itself) [20,21]. In the first instance, the movement of small ions, hydrophilic macromolecules, and water are controlled by tight junctions which are primarily composed of claudins, occludins, and junctional adhesion molecules and all play key roles in regulating the permeability of tight junctions [21]. On the other hand, the transcellular pathway shuttles a wide range of molecules, from (more hydrophobic) macromolecules such as lipoproteins and antibodies to smaller molecules such as amino acids, anions, and cations from one polar side to the other polar side of the endothelial cell using vesicles or receptor-mediated uptake and diffusion [22].

Furthermore, the endothelium can regulate blood flow and therefore vascular tone through the secretion of various vasoactive factors [13]. These vasoactive factors could be vasodilatory in nature such as nitric oxide (NO) and prostacyclin (PGI_2_), or vasoconstrictive such as endothelin-1 (ET-1) or thromboxane (TXA_2_). The effects these factors elicit are all involved in altering intracellular free Ca^2+^ concentration in vascular smooth muscle cells which in response leads to the constriction or dilation of the vessel wall.

Finally, another important role of the endothelium is the recruitment of leukocytes to a site of tissue injury during an inflammatory response [23]. Transendothelial migration (TEM) is a multistep adhesion cascade in which leukocytes, such as neutrophils, become progressively more adhesive to the endothelium involving a number of cell adhesion molecules such as selectins, integrins and their respective ligands [24,25]. The leukocytes then migrate through the endothelium via the paracellular route, whereby signalling events occurring on the interendothelial junctions lead to increased leukocyte trafficking caused by loosening the adhesive contacts of vascular endothelin cadherin (VE-cadherin) [24]. Alternatively, in a less common transcellular route, leukocytes pass directly through an individual endothelial cell, in a process involving the formation of a channel or pore [26].

### 1.2 The Dynamic Transcriptional Response of the Endothelium

Given its prominence in regulating key processes relating to vascular homeostasis, the endothelium must be able to react constantly and appropriately both to acute and chronic changes in environmental cues. Thus endothelial cells can effect rapid (and reversible) changes in vascular tone, permeability, coagulant activity and inflammation in response to stimuli from the circulation or microenvironment [27]. Post-translational mechanisms involving acute signalling pathways clearly play major roles in such homeostatic responses. For instance, the production of NO (and hence regulation of vascular tone), can be modulated acutely by phosphorylation of eNOS at multiple sites which are induced via signalling cascades activated by external cues such as changes in shear stress, intracellular calcium levels and ATP concentrations [28]. In addition, rapid and reversible changes in the endothelial cell transcriptome have been demonstrated to modulate function in response to, for example, hypoxia [29], hyperglycaemia [30] and disturbed shear stress [31]. In the case of hypoxia, these can be mediated by the well-documented, [O_2_]-dependent, post-translational changes in the stability and activity of the hypoxia-inducible (master transcriptional regulatory) factors (HIFs) [32,33]. In addition, endothelial cells can rapidly induce the transcription of other known master transcriptional regulators, such as peroxisome proliferator-activated receptor gamma coactivator-1 alpha (PGC-1*α*), a transcriptional coactivator that regulates energy metabolism and mitochondrial biogenesis [34,35] and Kruppel-like factor-2, a promoter of an anti-inflammatory and anti-thrombotic cellular phenotype. These are expressed in response to appropriate cellular cues, to alter the cellular transcriptome and hence function [36].

In addition to the modulation of activity/expression of such master transcription factors, gene transcription is in parallel regulated at the level of epigenetics. This relates to (heritable) changes in cellular phenotype which are independent of the primary genetic sequence. It encompasses the study of changes in the accessibility of chromatin and hence gene expression, mediated by covalent modifications to the DNA and histone proteins of chromatin and some non-coding RNAs [37,38].

## 2 Epigenetic Regulation of Gene Expression: DNA Methylation

Chromatin comprises a complex of genomic DNA and protein. The basic unit of chromatin organisation is the nucleosome in which DNA is wrapped around an octamer of histones [39]. Modifications to the histone proteins include phosphorylation, acetylation, methylation, and ubiquitination at diverse sites which act to alter the chromatin structure [38,40,41]. These modifications can either increase the affinity between DNA and histones (thus repressing gene transcription), or alternatively decrease the affinity, making the unit less condensed and making transcription more likely to occur. Histone acetylation, due to its occurrence on basic amino acids, is associated with gene activation as the basic charged amino acids are neutralised and weaken the affinity between DNA and histones [42]. Conversely, some other modifications, such as methylation of lysine 27 on histone H3, are strongly associated with gene silencing [43–45].

By contrast to the large (and ever-increasing) number of observed histone modifications, with diverse effects on gene transcription rates, the genomic DNA of eukaryotic chromatin is principally only subject to modification by methylation at cytosine-guanine (CpG) dinucleotides, which predominantly acts to repress gene transcription [37,46].

DNA methylation is catalysed by the family of DNA methyltransferases (DNMTs), whereby a methyl group (-CH_3_) is transferred from S-adenyl methionine (SAM) onto the 5th carbon base of the cytosine ring (C5) forming 5-methylcytosine (5-mC) [47]. Thus, DNMTs are termed epigenetic *writers*. Conversely, the enzymes involved in removing these methyl marks are known as *erasers*, whilst proteins that recognise and bind the modified DNA and modulate the activities of associated factors are termed *readers* [48].

Three family members of DNMTs are responsible for methylating DNA under different environmental conditions. DNMT1 is responsible for replicating DNA methylation patterns from the parent DNA strand onto newly-synthesised daughter strands during DNA replication, ensuring the preservation of these epigenetic marks across successive cell divisions, whilst DNMT3A and DNMT3B are commonly described as *de novo* DNMTs for their ability to create new methylation patterns on unmodified DNA [49]. As stated above, DNA methylation occurs almost exclusively on cytosine residues within cytosine-guanine dinucleotides [37]. Genomic areas with a rich CpG deposit are known as CpG islands and these islands are typically 200 bp in size and are found at promoter regions [44]. It is regarded as a modification that is repressive [37,46] and is considered to mediate its action by direct interference inhibition of transcription activator factor binding, or through the recruitment of specific transcriptional repressors to methylated DNA, such as methyl-CpG-binding proteins (MBPs), or by alteration of higher-order chromatin structure [46].

The MBPs are thus considered epigenetic readers [37, 50]. There are 11 members in this protein family and all of them share a common methyl-binding domain that enables them to recognise 5-mC. Generally, MBPs have been shown to play a role in maintaining higher-order chromatin structure and stability [51]. Thus, in addition to preventing the binding of transcription factors to their target sites, they can recruit histone deacetylases (HDACs) and other chromatin remodelling complexes leading to a closed chromatin state [52]. Accordingly, DNA demethylation plays a crucial role in gene regulation by counteracting the activities of DNMTs, thereby contributing to the level and specificity of DNA methylation [53].

DNA methyl marks can be removed passively or actively. Passive DNA demethylation occurs during cell division when DNMT1 is inhibited allowing cytosine to remain unmethylated such that over successive cell division cycles, these methyl marks gradually disappear [47]. Conversely, active DNA demethylation necessitates an enzymatic process to revert 5-mC to its native cytosine form. As the covalent bonds linking cytosine to the methyl group are exceptionally strong, active DNA demethylation involves a series of enzymatic reactions involving deamination of the amine group of the methylated cytosine residue or oxidation of the methyl group of 5-mC in order to transform it to a cytosine variant. To restore this cytosine variant to its native form, the cytosine undergoes base excision repair (BER) mechanisms [47]. Deamination can take place through the action of the activation-induced cytidine deaminase/apolipoprotein B mRNA-editing enzyme (AID/APOBEC) resulting in the conversion of 5-mC to thymine. This process generates a guanine/thymine (G/T) mismatch upon which the BER pathway then acts to restore the thymine to an original, unmodified cytosine [47]. Alternatively, oxidation of the methyl group is facilitated by a family of enzymes referred to as the ten-eleven translocations (TETs) which function as the erasers of epigenetic marks.

**Fig. 1:**
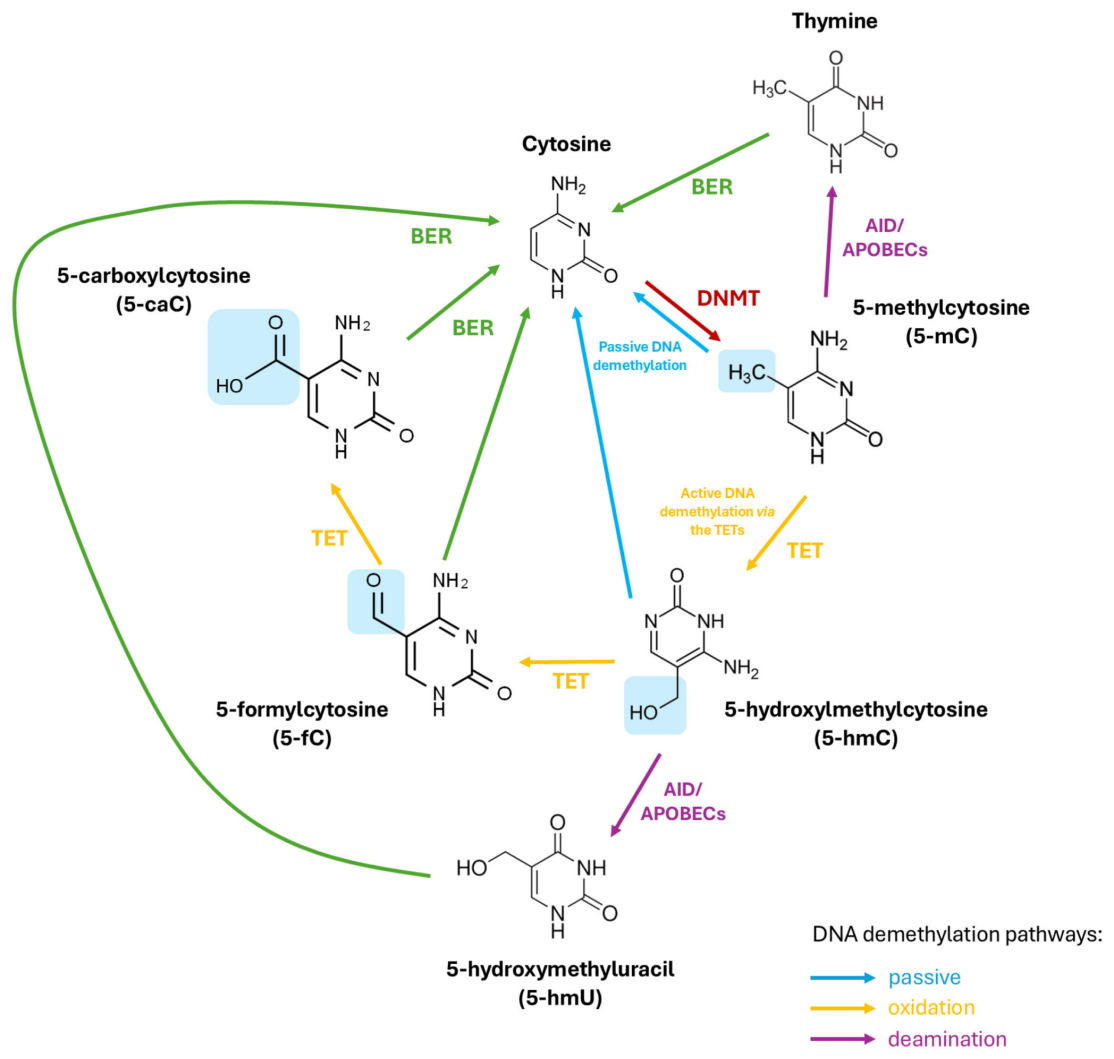
The (DNA) methylation and demethylation of cytosine. DNMTs add a methyl group (-CH_3_) to cytosine on CpG islands making 5-methylcytosine (5-mC). TET enzymes then successfully oxidise 5-mC to 5-hydroxylmethylcytosine (5-hmC), 5-formylcytosine (5-fC), and finally, 5-carboxylcytosine (5-caC) (via active demethylation). 5-mC and 5-hmC can also be converted back to cytosine passively through successive cell replication cycles. In an alternative deamination pathway, 5-mC or 5-hmC can be deaminated by activity-induced cytidine deaminase/apolipoprotein B mRNA editing complex (AID/APOBEC) deaminases to form thymine or 5-hydroxymethyluracil (5-hmU), respectively. Through base excision repair mechanisms (BER), 5-fC, 5-caC, 5-hmU, or thymine can be converted back to unmethylated cytosine. TET, ten-eleven translocation; DNMT, DNA methyl transferase; CpG, cytosine-guanine.

5-hydroxylmethlycytosine (5-hmC) was discovered in bacteriophage DNA in the 1950s [54], but its relevance in the mammalian genome remained unclear until the identification of the gene at the breakpoint of the ten-eleven translocations (t(10;11) (q22;q23)) found in myeloid leukaemia which was termed TET1. TET1 was found to have enzymatic activity, capable of converting 5-mC to 5-hmC [55]. It is now understood that 5-hmC is a key inter-mediate in the active, oxidation-dependent, demethylation pathway. In this pathway, the TET protein(s) catalyse the oxidation of 5-mC to 5-hmC through hydroxymethylation [56] and also mediate the successive oxidation of 5-hmC to 5-formylcytosine (5-fC), and finally to 5-carboxylcytosine (5-caC). The BER pathway, facilitated by thymine DNA glycosylase (TDG) [56], then replaces either 5-fC or 5-caC with the native form of cytosine, thus completely eliminating the methyl mark from cytosine [47] (Fig. 1). TETS are members of the 2-oxoglutarate-dependent dioxygenase superfamily and consequently require molecular oxygen (O_2_) and *α*-ketoglutarate (*α*-KG), (alternatively known as 2-oxoglutarate (2-OG)) as substrates and iron-2^+^ (Fe II) as a cofactor [57].

In mammals, the TETs comprise a family of 3 proteins: TET1, TET2, and TET3. Each of these TETs has a number of transcript and protein isoforms arising from differential splicing and the use of different promoters. Thus, in humans, 2 isoforms of TET1 have been identified, whilst 3 isoforms of TET2 and TET3 have been observed [58]. All three TETs are found to be widely expressed in all tissue types. However, each TET protein displays a different pattern of tissue- and developmental stage-specific expression, highlighting their distinct functions [59]. Within vascular cells, TET2, specifically, has been demonstrated to be of functional significance in regulating smooth muscle plasticity [60], and in regulating autophagy in endothelial cells [61].

Structurally, all three members possess a conserved core catalytic domain located at the C-terminus, consisting of two double-stranded *β*-helix (DS*β*H) containing metal-binding residues and a cysteine-rich domain situated on the N-terminus of the DS*β*H [62]. This cysteine-rich region helps stabilise TET-DNA interaction [62]. The DS*β*H also has binding sites for the TETs’ cofactors—Fe (II) and 2-OG. As a whole, the core catalytic domain is sufficient to facilitate catalytic activity [63]. At the N-terminus, there is a DNA-binding, zinc finger CXXC domain, which is present in TET1 and TET3 [56]. This domain aids in the enzyme’s ability to bind to CpG sequences in both methylated and unmethylated DNA to facilitate its catalytic activity [63]. On the other hand, TET2 does not possess a CXXC domain but interacts with CXXC finger protein 4 (CXXC4, also known as IDAX), which encodes a CXXC domain and is situated in a chromosomal location, adjacent to TET2 [56,64]. IDAX interacts directly with the core catalytic region of TET2 and binds to DNA sequences, facilitating the enzyme’s localisation to the promoter regions and CpG islands of genomic DNA [64].

## 3 DNA Methylation and CVD

Given its regulatory role in gene transcription, alterations in DNA methylation should lead to differential gene expression and consequently, changes in cellular phenotype [65]. Ever-increasing evidence suggests that aberrant DNA methylation associate with common diseases [66]. Further, epigenome-wide association studies (EWASs) of incident pathologies are beginning to identify predictive biomarkers of disease. Such findings both support a causal role for aberrant methylation in disease aetiology and shed light on the molecular mechanisms underlying disease progression [67,68]. This association of aberrant DNA methylation and human disease has been extensively studied in the cardiovascular field. Specifically in the case of large-artery AS, several retrospective studies, investigating associations between AS-related traits and levels of global methylation, methylation at candidate gene loci and epigenome-wide methylation have been undertaken and systematically reviewed [69,70]. To date, such studies have often given inconsistent results, due to the many confounding factors which can affect the epigenome, such as sex, ethnicity, age, lifestyle and socioeconomic status *etc*. Thus far, no consistent association between altered global levels of methylation (either hypo-or hyper-methylation) and CVD has become apparent. With regard to the candidate genes that have been investigated in more than one study (selected on the basis of previous genetic studies and/or biological function), consistent hypermethylation in the *ESRa, ABCG1* and *FOXP3* gene loci, and hypomethylation in the *IL-6* gene loci have been identified as being associated with CVD [69]. The direction of change of the methylation correlates with the altered gene expression (decreased expression from hypermethylated loci and increased expression from hypomethylated loci) that has been observed or might be predicted, in the diseased state [71–73].

The findings from unbiased, EWASs have been particularly hard to reproduce in replicate cohorts, due to both confounding factors as described above and (in most cases) their relatively small sample sizes. Nevertheless, an increasing number of differentially methylated loci have been identified in more than one EWAS, which correspond to genes relating to lipid and carbohydrate metabolism, inflammation and obesity [69]. A very recent systematic review of studies examining the associations of DNA cytosine methylation and CVD, including EWASs, has identified 1452 CpG sites mentioned in at least 2 publications, 441 CpG sites mentioned in at least 3 publications and 2 sites (proximal to the Zinc Finger Protein 438 and F2R Like Thrombin or Trypsin Receptor 3 genes) that were reported in at least 6 studies. With respect to mapped genes displaying differential methylation, 5807 have thus far been mentioned in at least 2 studies, with transcriptional enhanced associate (TEA) Domain Factor 1 and Protein Tyrosine Phosphatase Receptor Type N2 being the most frequently reported [74]. An easily accessible and searchable database has been created by aggregating all such CpG and gene-related information that can be used for future research. Thus, although further studies, involving larger sample sizes are needed to validate current findings and determine whether the changes in methylation are consistent with differential gene expression in CVD, there seems little doubt that the identification of differential DNA methylation associated with CVD will prove of great clinical value in the foreseeable future.

The overwhelming majority of these methylation studies analyse blood samples, due to the ease of sample collection, or (in some cases) bulk vascular tissue. Indeed, a recent study, interrogating differentially-methylated regions genes and differentially-expressed genes within atherosclerotic (compared to normal) aortic tissue, has revealed information regarding the enrichment of specific immune cell populations within the atherosclerotic plaque [75]. However, the challenge of correlating ED with altered methylation *specifically* within the *endothelium* in human studies *in vivo* is currently unrealistic. Nevertheless, there are many *in vitro* studies which link environmental cardiovascular risk factors to altered endothelial cell methylation and function, suggesting that the altered methylation may be causal in the cellular dysfunction.

## 4 Effects of CVD Risk Factors on Endothelial Cell Methylation *in Vitro*

Many of the independent CVD risk factors, associated with the sedentary lifestyle and overabundance of high-calorie food intake in modern society, comprise changes in blood plasma composition, such as hyperglycaemia, hyperinsulinemia, hyperlipidaemia (including hypercholesterolemia), hyperhomocysteinemia and hyperleptinemia [76–80]. The direct, pathogenic effects of such changes (as might be experienced *in vivo*) upon both microvascular and macrovascular endothelial cell function have, in many cases, been demonstrated *in vitro* [30,81–83].

Similarly, air pollution and smoking can reduce the oxygen-carrying capacity of the blood and result in exposure of endothelial cells to hypoxia, and in the accumulation of excessive reactive oxygen species (ROS), both of which are known to impact negatively upon endothelial cell function *in vitro* [84,85]. Chronic exposure to risk factors, such as high cholesterol and smoking, additionally exacerbates the susceptibility of the endothelium at bifurcations and curvatures of the arterial tree, to the deleterious effects of disturbed flow [86] as can also be observed *in vitro* [87].

Further, changes in global and/or locus-specific endothelial cell methylation, resulting from such exposure, as described above, have also been extensively studied in various endothelial cell types *in vitro*. For instance, in human aortic endothelial cells (HAECs), transiently exposed to different (patho)physiologically relevant levels of glucose, differentially-methylated genomic regions (DMRs) encompassing over 2000 gene loci have been identified. These DMRs included loci associated with a disproportionate number of genes involved in angiogenesis and nitric oxide signalling cascades. Further, the differential methylation at these loci was typically shown to correlate with altered expression of their associated gene transcripts [88]. The effects upon the endothelial cell methylome, arising from exposure to hyperglycaemia and other CVD-associated, environmental risk factors are summarised in Table 1 (Ref. [88–114]). These studies included investigation of the effects of hypoxia, altered blood flow, hyperhomocysteinemia, oxidative stress, inflammation, oxidised low density lipoprotein (LDL), and tobacco smoke and provide a framework for investigations of the molecular mechanisms underpinning aberrant DNA methylation and CVD risk factors. The associations of altered methylation with exposure to agents which cause cellular dysfunction clearly do not prove their functional role in such dysfunction (or, *in vivo*, in the associated disease). Nonetheless, the changes in the methylation patterns on specific gene loci involved in vascular homeostasis (notably eNOS; reviewed in 2021 [115]), together with the observed changes in transcriptional expression of such critical genes are clearly compelling. Further, treatments that reverse methylation (such as inhibition of DNMT) have often been shown to restore normal gene expression and endothelial cell function in these models, further highlighting the functional significance of the differential methylation [89–91,116].

### 4.1 Dysregulation of DNA Methylation by CVD Risk Factors: Potential Mechanisms

As described above, there is compelling evidence to link environmental risk factors, associated with CVD to altered endothelial cell methylation and dysfunction. Thus, the cellular cues, impacted upon the endothelium by these risk factors, must act to alter either the activity and/or specificities of the epigenetic writers or erasers. Functional changes in the activities of the epigenetic modifiers could result from changes in their expression and/or catalytic activity, while changes in specificity probably result from altered interactions with binding partners. Evidence for the causal effect on CVD development of genetic mutations (which could affect any of these properties) in TET2 and DNMT3A specifically comes from studies on clonal haematopoiesis of indeterminate potential (CHIP). CHIP refers to mutations in haematopoietic cells that lead to their clonal expansion [117]. It is associated with an increased risk of developing haematological malignancies such as leukaemia [118] and also CVD [119]. Intriguingly, the two most commonly mutated genes which give rise to CHIP are DNMT3 and TET2 [117,120]. Whilst this does not inform upon the specific role of altered DNA methylation in the endothelium, it is a clear demonstration of the functional significance of the altered expression and/or activities of these epigenetic modifiers in the development of CVD.

### 4.2 Effects of CVD Risk Factors on the Function of DNMTs

There is substantial evidence in the literature to suggest that environmental risk factors of CVD can alter DNMT expression and hence activity, and associate with an aberrant phenotype. Some of these studies are outlined in Table 2 (Ref. [121–130]). From these studies, it is evident that different observations are seen, depending on the cell type under investigation and whether it is *in vitro* or *in vivo* study. Nevertheless, it is clear that the expression of DNMTs is dynamic, tightly regulated and can be impacted by extracellular cues, although the precise mechanisms which underlie the changes in expression are not clear.

With regard to the (post-translational) regulation of activity, DNMTs use SAM as the methyl donor and are strongly inhibited by S-adenosylhomocysteine (SAH), which is produced as a result of this donation [131]. An altered SAM/SAH ratio and hyperhomocysteinemia (which typically results in elevated SAH levels) are both considered risk factors for AS development [79,132] and likely act via inhibition of DNMT activity. Studies have also shown that both the activities and specificities of the DNMTs are modulated by cellular redox. Thus in the absence of SAM and in a pro-oxidant chromatin microenvironment (as, for instance, might be induced by smoking, hyperglycaemia or air pollution *etc*. [133]), DNMT3A/B may act to convert 5-mC or 5-hmC to unmodified cytosine [134], while oxidative damage of O^6^-methylguanine can inhibit DNMT binding, leading to hypomethylation [135]. Localised oxidative DNA damage, induced by H_2_O_2_ treatment, was also shown to recruit DNMT1 to sites of damaged chromatin and induce the formation of complexes (including DNMT3B), and thus act to relocalise DNA methylating, transcriptional-silencing activity to aberrant sites [136]. Some other studies, linking changes in DNMT activity to CVD risk factors, are also included in Table 2.

### 4.3 Effect of CVD Risk Factors on the Function of TETs

As is the case for DNMT expression, many studies have demonstrated the (transcriptional) expression of TETs to be dynamic and to be impacted by CVD environmental risk factors. Various modifiable environmental risk factors such as a diet rich in fat and the resulting change in blood flow, alcohol, smoking, as well as air quality have been shown to influence the expression of the TETs *in vivo*. Again, as was the case for DNMTs, conflicting results have been reported, which may reflect different experimental conditions. Several studies have also shown effects on TET2 transcription, specifically in endothelial cells, *in vitro*, resulting from altered shear stress and administration of oxidised low density lipoprotein (LDL). A brief overview showing the associations of different risk factors to TET expression is outlined in Table 3 (Ref. [137–150]).

### 4.4 Changes in TET Activity Due to Substrates and Co-Factors

As stated previously, as members of the 2-oxoglutarate-dependent dioxygenase superfamily, the catalytic activities of TETs are dependent on molecular oxygen and a-ketoglutarate as substrates and Fe(II) as a co-factor [56,57]. Further, their activities are inhibited by steric competition of a-ketoglutarate by the downstream tricarboxylic acid (TCA) metabolites, succinate and fumarate and the oncometabolite 2-hydroxyglutarate (2-HG) [151,152], and by accumulation of the more oxidised iron cation, Fe(III) [153]. Thus, hypoxia, cellular metabolism and oxidative stress can all potentially affect the catalytic activities of TETs. Indeed, the effects of cellular oxygen availability, metabolic flux and redox have all been shown empirically to affect TET activity, using 5-hmC as a surrogate marker of that activity [154–156]. As has been discussed above, these cellular states are all known to be impacted by lifestyle risk factors associated with CVD. For instance, hyperglycaemia, associated with diabetes, can potentially change oxygen availability, metabolic flux and cellular redox [157], while smoking, hyperlipidaemia, and hyperhomocysteinemia are well-documented to be associated with oxidative stress [158,159]. In addition, the catalytic requirement of TETs for Fe(II) renders them susceptible to diminished levels of vitamin C, which is required to recycle this more reduced form from Fe(III) [160] and intake of vitamin C is highly dependent on nutrition. Moreover, smokers have been shown to have depleted levels of vitamin C [161], further suggesting mechanistic associations between some CVD risk factors and altered TET activity. Changes in 5-hmC levels, taken as a measure of TET activity have been observed in both blood and solid organs as a result of acute glucose administration *in vivo* [162,163] while peripheral blood mononuclear cells (PBMCs) collected from diabetic patients were found to display decreased levels of 5-hmC [164]. Altered 5-hmC levels in lung cells *in vivo* are also found to be associated with smoking-related cancers [165] and 5-hmC signatures in circulating cell-free DNA show the potential to become diagnostic and predictive biomarkers for coronary artery disease [166]. However, it should be noted that these changes in 5-hmC levels may result as a consequence of both altered TET expression and/or activity in the disease states.

### 4.5 Post-Translational Modifications

TETs are increasingly being demonstrated to be regulated by a number of known post translational modifications (PTMs) which include phosphorylation, methylation, acetylation, PAPylation, GlcNAcylation, and ubiquitination [58,167]. Such PTMs can regulate both TET specificity and functional activity via modulation of, for example, their catalytic activity, stability, interactions with other chromatin-associated proteins, and DNA-binding capability. Furthermore, these can be affected by CVD risk factors such as hyperglycaemia. For instance, TET2 stability and activity have been shown to be regulated by phosphorylation at serine 99, mediated by AMP-activated kinase (AMPK) [164]. AMPK is both a cellular metabolic and redox sensor [168] and therefore high glucose and/or altered cellular redox could act to alter TET2 activity via an AMPK-dependent mechanism to account for the decreased levels of 5-hmC observed in the PBMCs of diabetics [169]. All three TET proteins are also known to interact strongly with the *O*-linked GlcNAc transferase, which adds a GLcNac group to serine and threonine residues of TET proteins and thereby regulates TET function by decreasing the number of available phosphorylation sites [170]. This *O*-linked GlcNAC posttranslational modification is highly regulated and linked to cellular metabolism as the donor sugar for *O*-GlcNAcylation (UDP-GlcNAc) is synthesized from glucose, glutamine, and UTP via the hexosamine biosynthetic pathway [171] and dysregulated *O*-GlcNAcylation has been linked to diabetes [172]. In addition, *O*-GlcNAc modification is increased under levels of oxidative stress [173], again suggesting dysregulation of *O-*GlcNAcylation as a potential mechanism to link CVD risk factors to altered TET function and DNA methylation.

Oxidative stress can also influence acetylation of TET2, such that (by contrast to the situation described above in section 4.4) it *increases* TET2 activity and stability. This was demonstrated by the addition H_2_O_2_ to human ovarian carcinoma (A2780) cells which resulted in increased global 5-hmC levels. Further investigations revealed that TET2 activity increased due to stress-induced p300-mediated TET2 acetylation, resulting in the enhanced binding to DNMT1. This tight binding to DNMT1 (and chromatin) protected against accumulation of abnormal DNA methylation (typically induced by oxidative stress) by converting 5-mC to 5-hmC [174]. This seemingly paradoxical effect of oxidative stress on TET activity may be context dependent (due to differences in cell type and/or species of ROS) or may reflect differences in adaptive *versus* pathological cellular responses to changes in cellular redox.

These are just a few examples highlighting how some of the risk factors of CVD could indirectly affect TET function via altering their post-translational modifications, and it is clear that these are complex relationships about which there remains much to discover.

## 5 Concluding Remarks

Epigenetic changes are now strongly believed to link so-called “modifiable” risk factors, associated with an unhealthy lifestyle. to the development of CVD. Further, aberrant DNA methylation, in particular, as a regulator of gene transcriptional expression, is increasingly believed to not only associate with, but to be causal in, the cellular dysfunction that characterises both the aetiology and progression of CVD. Dysfunction of the endothelium is likely to be one of the earliest steps in the development of AS, a common hallmark of most CVD, and therefore understanding how environmental risk factors might act to alter the methylome of the arterial endothelial cells is crucial to understanding the aetiology of CVD. As discussed here, the methylome of the endothelium, and changes associated with CVD *in vivo* are challenging to demonstrate. Nevertheless, aberrant DNA methylation and changed transcriptional expression in critical genes linked to endothelial cell function and homeostasis have been demonstrated *in vitro* by exposure to known risk factors.

**Fig. 2:**
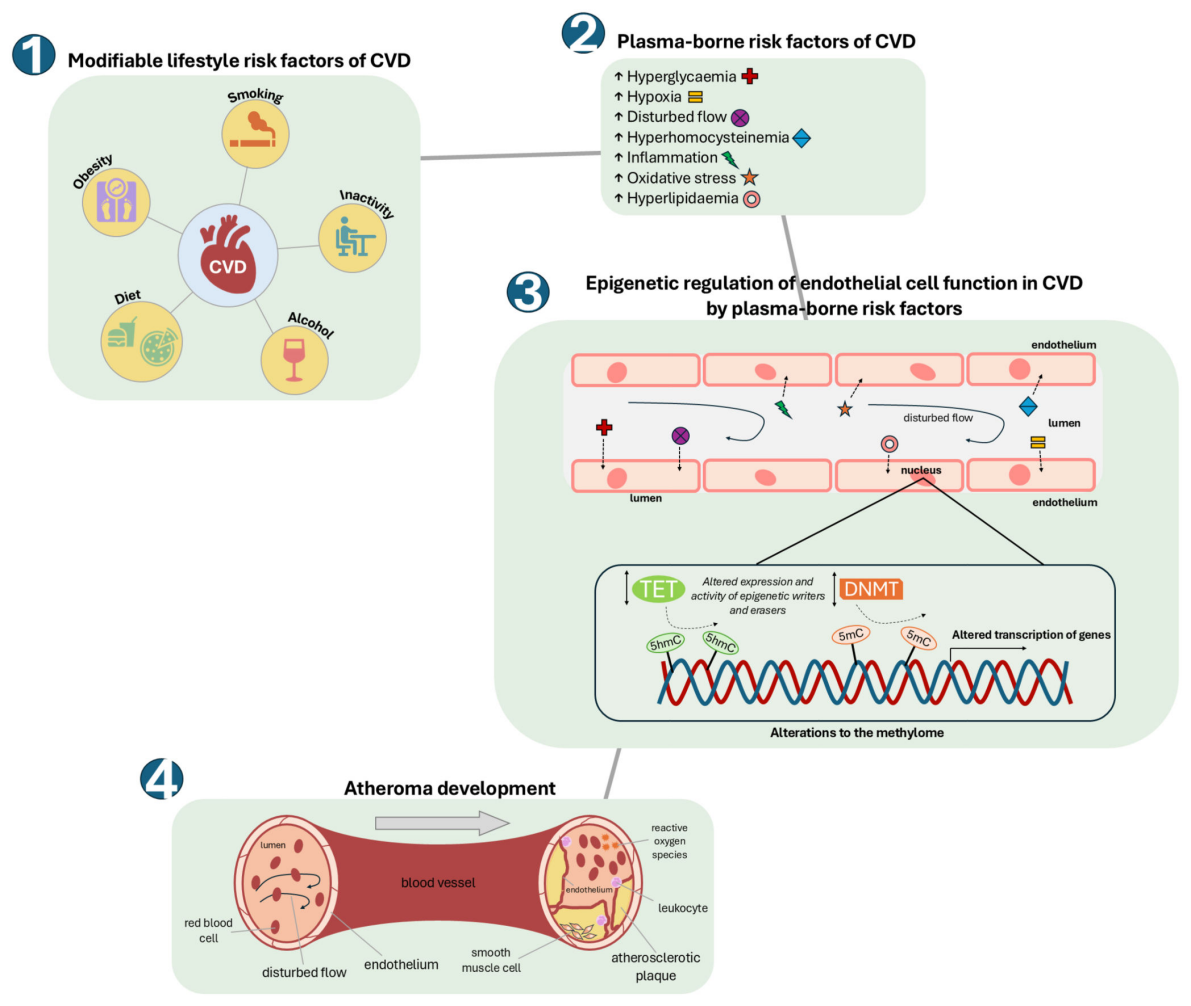
How modifiable risk factors of CVD can alter the endothelial cell methylome, leading to the development of atherosclerosis. An unhealthy ‘Western lifestyle’ can lead to significant changes in plasma levels of modifiable risk factors of CVD such as hyperlipidaemia (including hypercholesterolemia), hyperglycaemia and hyperhomocysteinemia, together with lower available molecular oxygen (hypoxia), increased reactive oxygen species (oxidative stress) and alterations in blood flow. These changes can impact upon the activities and/or specificities of epigenetic modifiers (TETs and DNMTs) within the cells of the endothelium to alter the endothelial cell methylome and transcriptome. This in turn can result in endothelial dysfunction and consequent development of atherosclerosis.

We have explored here the evidence that these risk factors can alter epigenetic marks by modulating the expression and activities of the epigenetic erasers and writers, to highlight possible molecular mechanisms which might be critical in disease aetiology (summarised graphically in Fig. 2). It is also increasingly becoming evident that the specificity of these epigenetic modifiers may be mediated by their many binding partners [58,59]. Although not fully addressed here, the expression/activity/availability of a specific binding partner might, of course, also be subject to modulation by the known environmental risk factors and this will, no doubt, be a topic of future investigations.

The millions of methylation sites within the human methylome together with the complex interactions involved in the pathology of ED and CVD make the combination of artificial intelligence and network medicine a certain and powerful future approach to understanding the role of the methylome in CVD. By integrating methylation data with other epigenetic data, together with transcriptomic and functional genomic data, the construction of disease models, dependent upon methylation at key regulatory regions will become possible [175,176]. The identification of specific methylation marks, that are characteristic and/or causal in disease together with elucidation of the molecular mechanisms underlying the development of the pathology, would clearly inform the development of diagnostic and predictive biomarkers and novel therapeutics. However, it should also be noted that aberrant epigenetic marks are dynamic and are increasingly being demonstrated to be reversible by environmental factors associated with a healthy lifestyle, such as exercise and cessation of smoking [177,178]. Therefore, greater emphasis and importance should also perhaps be given to public health campaigns which encourage healthier lifestyles, in both the prevention and treatment of CVD [179].

## Figures and Tables

**Table 1:** *In vitro* studies showing the association between aberrant DNA methylation and cardiovascular disease (CVD) risk factors in endothelial cells.

Risk factor	Experimental design	Cell model and conditions	Hypo-/Hyper-methylation?	Conclusions/Outcomes	References
Hyperglycaemia	Genome-wide DNA methylation quantification	Cultured human endothelial aortic cells (HAECs); diabetic (250 mg/dL), pre-diabetic (125 mg/dL), euglycemic (100 mg/dL) glucose concentrations for 72 hrs	Hypomethylation of *NOS3* Hypermethylation of *VEGF*	Endothelial cells encode a glycaemic memory Molecular pathways associated with angiogenesis are most statistically enriched	Pepin *et al.,* 2021 [88]
Hyperglycaemia	Bisulfite sequencing of CXCR4 promoter	Cord-blood-derived CD34^+^ stem cells; mannitol 30 mM or glucose 30 mM until cells lost glucose tolerance then recovered in normoglycemic conditions for 3 days	Hypermethylation of CXCR4 promote	Negatively affects migration ability towards SDF-1*α* Hallmarks of glycaemic memory after 3-day recovery in normoglycemic conditions	Vigorelli *et al*., 2019 [92]
Hyperglycaemia	Promoter DNA methylation assessed by bisulfite sequencing	Human umbilical vein endothelial cells (HUVECs) treated with low (5 mM) and high (25 mM) glucose for up to 48 hrs	Expression of *ET-1* gene was upregulated in high glucose, but no change in methylation status at the promoter site	Increased expression of *ET-1* gene may be in part due to increased oxidative stress	Binjawhar *et al.,* 2023 [93]
Hyperglycaemia	Promoter DNA methylation patterns of the protein p66^Shc^ adaptor protein investigated using methylation-sensitive or dependent enzymes	HAECs were exposed for 6 days to either normal (5 mmol/L) or high (25 mmol/L) glucose concentrations then to high glucose for another 3 days then to low glucose for a final 3 days	Hypomethylation of CpG dinucleotides following high glucose treatment	Glycaemic memory as hypomethylation of p66^Shc^ did not change after normoglycemia was restored	Paneni *et al*., 2012 [94]
Hyperglycaemia	Genome-wide bisulfite sequencing analysing >750,000 CpG sites	Human retinal microvascular endothelial cells (HRECs) and HUVECs were treated with either 5 mM or 25 mM glucose for 2 or 7 days	Predominant DNA hypermethylation 88 differentially methylated regions (DMRs) for HUVECs and 8 for HRECs These DMRs are involved in the regulation of embryonic development (e.g., *HOX*) and cellular differentiation (e.g., *TFG-β)*	Increased hypermethylation observed in long-term culture compared with short-term culture Cell culture duration is a strong and more significant inducer of DNA methylation compared with glucose stimuli alone	Aref-Eshghi *et al.,* 2020 [95]
Hyperglycaemia	Methylation analysis of *SIRT6* was investigated by pyrosequencing- based methylation analysis	HAECs were exposed to either 5.5 mM (normal) or 30 mM (high) glucose for 7 days followed by 7 days of normal glucose	Hypomethylation of *SIRT6* promoter in cells exposed to high glucose compared to normal glucose	Epigenetic change induced by hyperglycaemia may be related to the regulation of TET2 expression (which increased) SIRT6 plays an important role in glucose homeostasis and metabolic disease; has been shown to be protective against endothelial dysfunction by being involved in the DNA-damage repair system	Scisciola *et al.,* 2021 [96]
Hypoxia	Methylation level of *DRP1* promoter region was detected using primers for methylation and unmethylation of the DRP1 promoter	Human pulmonary artery endothelial cells (HPAECs) maintained at 37 °C, 5% CO_2_ and 100% relative humidity or water-saturated atmosphere comprising 3% O_2_, 5% CO_2_ and 91% N_2_ (mimicking hypoxic conditions) for 24 hrs	Hypomethylation of *DRP1* promoter under hypoxic conditions	Upregulation of *DRP1* is involved in pulmonary vascular remodelling in hypoxic pulmonary hypertension	Wang *et al.,* 2022 [97]
Hypoxia	MeDIP-qPCR used to detect methylation of *RASAL1* promoter	Human coronary artery endothelial cells (HCAECs) were maintained in a hypoxic-like environment for 72 hrs (1% O_2_, 98% N_2_)	Hypermethylation of *RASAL1*	Hypoxia induces EndMT through different pathways, one of which involved DNMT3A-mediated hypermethylation of *RASAL1* leading to constitutive Ras hyperactivity	Xu *et al*., 2016 [98]
Hypoxia	Bisulfite sequencing of the promoter region of human *miR-21*	Human umbilical artery endothelial cells (HUAECs) exposed to normoxia (5% O_2_) or hypoxia (1% O_2_) for 48 hrs	Hypermethylation of *miR-21* gene promoter site in hypoxic HUAECs	HUAECs exposed to hypoxia showed a transient increase in eNOS and DDAH1 which may be in part due to *miR-21*	Peñaloza *et al.,* 2020 [99]
Disturbed flow	Differential CpG site methylation of *KLF4* was measured by methylation-specific PCR, bisulfite pyrosequencing, and restriction enzyme PCR	HAECs were subjected to pulsatile undisturbed flow or oscillatory disturbed flow containing a flow-reversing phase	Hypermethylation of CpG islands within the *KLF4* promoter under disturbed flow	Haemodynamics influence endothelial *KLF4* expression. Downregulation of *KLF4* is associated with atherosusceptibility	Jiang *et al.,* 2014 [100]
Disturbed flow	Endothelial cells were stained with 5-mc antibody and visualised by epi-fluorescence microscopy DNMT1 expression was also quantified using qPCR	HUVECs were subjected to either pulsatile (athero-protective) or oscillatory shear (athero-prone) stress using a parallel plate circulating flow chamber for 24 hrs	Hypermethylation of the nuclei of endothelial cells subjected to oscillatory shear force	Oscillatory shear induces expression of DNMT1 expression and is found to mediate the hypermethylation observed in endothelial cells Atheroprone hemodynamic forces act as a risk factor for atherosclerosis	Zhou *et al.,* 2014 [101]
Disturbed flow	Reduced representation bisulfite sequencing (RRBS) and microarray analyses to investigate global DNA methylation patterns 5-aza and siRNA treatment of HUVECs to understand DNMT enzymatic activity	HUVECs were subjected to either unidirectional flow (laminar shear stress) or bidirectional flow (oscillatory shear stress) for 24 hrs	Hypermethylation of *HoxA5, Tmem184b, Adamtsl5, Klf3, mklr1, Pkp4, Acvrl1, Dok4, Spry2,* and *fp46* genes at the promoter region	DNMT1 was enhanced by oscillatory shear stress and a reduction in DNMT activity resulted in reduced OS-induced endothelial inflammation *HoxA5* and *Klf3* could serve as a mechanosensitive master switch in gene expression	Dunn *et al.,* 2014 [89]
Hyperhomocy-steinemia (Hcy) v	*LOX-1* DNA methylation changes rere examined using nested touchdown methylation-specific PCR (ntMSP) analysis	CRL-1730 endothelial cells were ntreated with either Hcy at different concentrations (0–500 μmol/L) or 100 μmol/L Hcy with 30 μmol/L vitamin B_12_ and 30 μmol/L folic acid for 72 hrs	Hypomethylation of LOX-1 in the Hcy-treated only groups	Hcy decreased the levels of DNMT1 Hcy increased levels of NF-*κ*B which promotes the transcription of target genes; NF-*κ*B is also involved in the pro-inflammatory response	Ma *et al,* 2017 [102]
Hyperhomocy-steinemia (Hcy)	CpG methylation in the human p66^Shc^ promoter region was quantified by methylation-specific real-time PCR using bisulfite-converted genomic DNA	HUVECs were treated with 10–200 μM Hcy for 48 hrs	Hypomethylation of CpG (6,7) in the p66^Shc^ promoter	Hcy upregulates p66^Shc^ expression and is important in homocysteine-induced endothelial cell dysfunction The effect of Hcy on the methylation status of CpG dinucleotides is gene- and site-specific	Kim *et al*., 2011 [103]
Hyperhomocy-steinemia (Hcy)	Bisulfite genomic DNA sequencing DNA methyltransferase (DNMT) activity assay	HUVECs were treated with 50 μM DL-Hcy for 48 hrs	Hypomethylation of 2 cytosine residues in the cyclin A CpG island	Hcy inhibits DNMT1 activity Hcy inhibits cyclin A transcription via hypomethylation-related mechanism leading to growth inhibition in endothelial cells; cyclin A promotes G1/S and G2/M transitions of the cell cycle	Jamaluddin *et al.,* 2007 [104]
Hyperhomocy-steinemia (Hcy)	Methylation-specific polymerase chain reaction (MS-PCR) was used to examine DNA methylation level of *DDAH2*	HUVECs treated with Hcy (0.1, 0.3, 1, 3, 190, 30 mM) for 48 hrs	Hypermethylation of two CpG islands located in the promoter of *DDAH2* in HUVECs treated with Hcy	Inhibition of DNMT1 using 5-aza prevented *DDAH2* gene promoter from DNA hypermethylation DDAH2 is a key enzyme for degradation for ADMA, ADMA is an endogenous inhibitor of NOS and can induce apoptosis of endothelial cells via increased oxidative stress	Jia *et al*., 2013 [90]
H_2_O_2_ (ROS)	hMeDIP-qPCR analyses were carried out to detect methylation level of CpG islands of the *ZO-1* promoter	HUVECs were treated with 10 μM H_2_O_2_ for 6 hrs	Hypermethylation of CpG islands in the promoter region of the *ZO-1* gene following oxidation with H_2_O_2_	H_2_O_2_ significantly decreased the expression of 5-hmC and ZO-1, a tight junction protein TET activity was also found to be essential in regulating *ZO-1* expression	Wang *et al*., 2022 [105]
Inflammation	Global DNA methylation was measured using a microarray	HUVECs were treated with 20 ng/mL TNF-*α* for 24 hrs	Hypomethylation of (223) genes associated with autoimmune/inflammation, infectious and cardiovascular disease, and cancers	Several of the hypomethylated genes were located at the promoter regions	Rhead *et al*., 2020 [106]
Inflammation	Bisulfite sequencing of *KLF2* promoter	Primary human umbilical vein endothelial cells (HUVECs) were treated with 0–2 μg/mL lipopolysaccharide (LPS) for 24 hrs	Hypermethylation at 12 CpG sites within the *KLF2* promoter	Methylation of *KLF2* modulated changes to eNOS and thrombomodulin, both of which confer potent anti-thrombotic and anti-inflammatory properties These changes may be in part mediated by increased DNMT1 activity	Yan *et al*., 2017 [107]
Inflammation	Chromatin immunoprecipitation (ChIP) analysis	HUVECs were treated with 10 ng/mL TNF-*α* for 48 hrs	Hypermethylation in two distinct regions within the ACE promoter	Effects may be mediated by a transient decrease in DNMT3A and DNMT3B but not DNMT1 TET1 protein expression was downregulated DNA methylation decreased the binding affinity of the transcription factor X to the ACE promoter	Mudersbach *et al.,* 2019 [91]
Hyperlipidaemia (Ox-LDL)	Global DNA methylation status was determined using a [^3^H] dCTP extension assay Gene-specific promoter methylation analysis using bisulfite sequencing	HUVECs were treated with 0, 20, 40 μg/mL ox-LDL for 24 hrs at passages 0, 3, and 5	Global hypermethylation following acute ox-LDL treatment Hyper- and hypo-methylation at the promoter regions of individual genes observed	Initial challenge of Ox-LDL causes an increase in global DNA methylation but displays a resistance to development of apoptosis upon subsequent exposure to ox-LDL Effects of exposure to ox-LDL appear to be mediated by activation of LOX-1 by reducing NO synthesis, enhancing coagulation pathways, and inducing endothelial cells apoptosis	Mitra *et al*., 2011 [108]
Hyperlipidaemia (Ox-LDL)	Global levels of DNA demethylation were assessed using 5-hmc antibody for immunofluorescence staining	HUVECs were pretreated with 5 mM total reactive oxygen species (ROS) scavenger N-acetyl-l-cysteine (NAC) and 20 μM nuclear factor-*κ*B (NF-*κ*B) inhibitor (BAY 11–7082) for 2 hrs before subsequently treated by 100 μg/mL ox-LDL for 24 hrs	Hypo-demethylation following ox-LDL treatment	Downregulation of TET2 and increasing NF-*κ*B activation was observed in ox-LDL induced pyroptosis in vascular endothelial cells	Zhaolin *et al*., 2019 [109]
Hyperlipidaemia (Ox-LDL)	DNA methylation status of *KLF2* promoter region was examined using chromatin immunoprecipitation (ChIP) assays	HUVECs were incubated with 200 μg/mL ox-LDL for 8 hrs	Hypermethylation of endothelial-*KLF2* following ox-LDL treatment	Ox-LDL represses endothelial-*KLF2* expression via DNA and histone methylation Downregulation of *KLF2* leads to endothelial dysfunction	Kumar *et al.,* 2013 [110]
Hypercholester-olemia	CpG methylation was quantified by methylation-specific real-time PCR using bisulfite-converted genomic DNA	HUVECs were treated 0, 50, 100, 200, 500 μg/mL LDL for 24 hrs	Hypomethylation of two CpG dinucleotides and acetylation of histone 3 in the human *p66shc* promoter following LDL treatment	The two CpG dinucleotides mediate LDL-stimulated p66shc promoter activity P66shc mediates LDL-stimulated increase in expression of ICAM-1 and decreased expression of thrombomodulin P66shc is involved in LDL-stimulated adhesion of monocytes to endothelial cells and plasma coagulation	Kim *et al*., 2012 [111]
Smoking	The methylation status of the *Bcl-2* promoter was observed by PCR amplification and sequencing of bisulphite converted DNA	HUVECs were treated with cigarette smoke extract (CSE) or CSE and 5-aza-2’-deoxycytidine (5-aza), or 5-aza and PBS for 12 hrs	Hypermethylation of *Bcl-2* promoter region after CSE treatment	The interactions between anti-apoptotic B-cell lymphoma-2 (Bcl-2) and pro-apoptotic Bcl-2-associated X (Bax) protein determines whether the mitochondria will release cytochrome c (cyc C), which is the initial factor for apoptosis Inhibiting *Bcl-2* promoter methylation from cigarette smoke-induced aberrant methylation may prevent endothelial apoptosis	Zeng *et al,* 2020 [112]
Smoking	CpG methylation status was determined by methylation-specific PCR and direct bisulfite sequencing	HUVECs were treated with 2.5% cigarette smoke extract (CSE) for 24 hrs after pretreatment with 1 μM 5-aza for 48 hrs	Hypermethylation of *COXII*	CSE induces aberrant *COX II* methylation and subsequent apoptosis in HUVECs COX dysfunction is common in disease	Yang *et al*., 2015 [113]
Smoking	Sequencing of bisulfite converted, PCR-amplified was carried out to understand the methylation status of CpG islands in the *mtTFA* promoter region	HUVECs were treated with cigarette smoke extract (CSE) or 1 μM 5-aza for 24 hrs	Hypermethylation of *mtTFA*	Mitochondrial transcription factor A (mtTFA) regulates mitochondrial transcription initiation and thereby, its function Inactivation of mtTFA promoter may contribute to the development and progress of COPD through cigarette smoke	Peng *et al*., 2019 [114]

CXCR4, C-X-C motif chemokine receptor 4; SDF1*α*, stromal cell-derived factor 1 alpha; CpG, cytosine-guanine; TET2, ten-eleven translocation -2; SIRT6, sirtuin 6; DRP1, dynamin-related protein 1; DNMT3A, DNA methyltransferase 3 alpha; eNOS, endothelial nitric oxide synthase; DDAH1, dimethylarginine dimethylaminohydrolase 1; ADMA, asymmetric dimethylarginine; ZO-1, zonula occludens-1; TNF-*α*, tumor necrosis factor alpha; ACE, angiotensin I converting enzyme; dCTP, deoxycytidine triphosphate; COX, cyclooxygenase; COPD, chronic obstructive pulmonary disease.

**Table 2:** Environmental risk factors of CVD and their impact on DNMT expression and activity.

Risk factor	DNMT expression level	Effect/possible causes	Reference
Tobacco smoke	Increased activity of DNMT1 in adenocarcinoma cells of smokers compared to non-smokers	Increased hypermethylation at the promoter region of tumour suppressor genes important in the pathogenesis of squamous cell carcinoma	Kwon *et al.,* 2007 [121]
Alcohol consumption	Decreased DNMT3A and DNMT3B protein activity in patients with alcoholism compared to healthy controls in peripheral mononuclear cells	Despite a decrease in DNMT3 activity, there was increased global DNA methylation in patients with alcoholism, but this was due to elevated homocysteine	Bonsch *et al*., 2006 [122]
Alcohol consumption	Increased dose of ethanol increases DNMT1 and DNMT3A mRNA in amygdala and bed nucleus of the stria terminalis tissues of rats	This increase may be in part a compensatory mechanism to the decrease in DNMT1 protein activity	Sakharkar *et al.,* 2014 [123]
Hyperglycaemia	Increased mRNA expression of DNMT3B in type 2 diabetes in HUVECs	Increased oxidative stress in T2D could facilitate activation of DNMT3B and subsequently, hypermethylation leading to an aberrant phenotype	Sultan and AlMalki, 2023 [124]
Hyperglycaemia	Decreased DNMT1 and DNMT3A expression levels following three months of hyperglycaemia in insulin deficient Ins2^Akita^ mice	Increased oxidative stress (high ROS production) led to decreasing DNMT1 and DNMT3A activity	Maugeri *et al.,* 2018 [125]
Hyperlipidaemia	Increased DNMT3B expression in the presence of ox-LDL in HUVECs	Increased DNMT3B activity led to a suppression of cellular repressor of E1A-stimulated genes (CREG), a vascular-protective molecule in atherosclerosis	Liu *et al*., 2020 [126]
Excess sodium intake	Increased DNMT3A expression in salt-sensitive rats	Increased DNA *de novo* (de)methylation in the kidney which contributed to the development of hypertension	Liu *et al*., 2018 [127]
Nutrient intake	Polyphenol-rich extracts (fruit and peel extracts) reduced the expression of DNMT1 and DNMT3A in both the kidneys and liver	Reducing DNMT activity may delay malignant cell growth progression	Nowrasteh *et al*., 2023 [128]
Exercise	Acute exercise reduced the expression of DNMT3A and DNMT3B in leukocytes	Exercise triggered global hypomethylation and increased mRNA expression of PPARGC1A. PPARGC1A upregulated mitochondrial biogenesis	Hunter *et al*., 2019 [129]
Disturbed flow	Low and oscillating shear stress induces DNMT1 expression and DNMT-dependent genome-wide DNA methylation patters	11 mechanosensitive genes such as *HoxA5* and *Flf3* were hypermethylated in their promoter region Methylation of cAMP-response elements (CRE) may act as a mechanosensitive master switch in gene expression	Dunn *et al*., 2015 [130]

T2D, type 2 diabetes; PPARGC1A, peroxisome proliferator-activated receptor gamma coactivator 1-alpha.

**Table 3:** Environmental risk factors of CVD and their impact on TET expression.

Risk factor	TET expression level	Effect/possible causes	Reference
High-fat diet	Increased expression of TET3 in the hearts of mice fed a high fat diet	Global changes to DNA hydroxymethylation have been shown to lead to cardiac hypertrophy e.g., *Mef2C* and *Xirp2*	Ciccarone *et al.,* 2019 [137]
High-fat diet	Decreased expression of TET1, TET2, TET3 in mice on a high fat diet	Increased ratio of (succinic acid + fumaric acid)/*α*-ketoglutarate *MEF2C* upregulated, increased cardiac hypertrophy	Pei *et al*., 2024 [138]
Ox-LDL	Administration of ox-LDL downregulates TET2 expression in HUVECs	Downregulation of TET2 led to the downregulation of the CSE/H_2_S system, which serves as a protective mechanism against the development of atherosclerosis The CSE/H_2_S system can inhibit NF-*κ*B and attenuate the adhesion of THP-1 cells to ox-LDL-activated HUVECs	Peng *et al*., 2017 [139]
Disturbed flow	Low shear stress down-regulates TET2 expression	Down-regulation of TET2 promoted endothelial mesenchymal transformation (EndMT)	Li *et al*., 2021 [140]
Disturbed flow	TET2 expression was downregulated under low shear stress in endothelial cells	Low shear stress downregulated genes involved in endothelial cell autophagy (important in maintaining cell health), namely *BECLIN1* and *LC3II,* by impaired TET2 expression thus contributing to the atherogenic process	Yang *et al*., 2016 [141]
Disturbed flow	Low shear stress inhibited endothelial cell TET2 expression	Upregulated succinate dehydrogenase B (SDHB) expression by decreasing recruitment with HDAC2 Increased SDHB leads to mitochondrial dysfunction, increases ROS generation, and pyroptosis	Chen *et al*., 2021 [142]
Smoking	Increased mRNA expression of TET1 but not in TET2 or TET3 following exposure to cigarette smoke extract (CSE) for 24 hrs in A549 cells	Dysregulated activity of TET enzymes has been identified as a causative mechanism in cancer TET1 (and TET2) play a crucial role in regulating the production of cytokines/chemokines in response to CSE challenge	Kaur and Batra, 2020 [143]
Smoking	Decreased expression of TET2 in smokers	*TET2* was among six genes identified from a genome-wide analysis in individuals with low values of forced expiratory volumes. These genes were associated with the development of chronic obstructive pulmonary disease Suggests genetic predisposition plays a significant role in the development of lung diseases	Wain *et al*., 2015 [144]
Physical (in)activity Alcohol consumption	Hippocampal and hypothalamic TET1 and TET2 mRNA expression was increased after voluntary exercise Increased TET1 mRNA expression in the cortex of psychotic (PS) patients comorbid with chronic alcohol abuse	Higher hippocampal 5-hmC content in the promoter region of *miR-137,* a miRNA involved in adult neurogenesis Exercise improved memory Alcohol-associated global DNA hypomethylation is observed in several forms of cancer and other pathological conditions A reduction of SAM was reported in the brain of alcoholic subjects	Jessop and Toldeo-Rodriguez, 2018 [145] Guidotti *et al*., 2013 [146]
Alcohol consumption	Increased expression of TET1 in nucleus accumbens following chronic intermittent ethanol (CIE)	Ethanol-induced epigenetic regulation altered behaviour c, accentuated withdrawal symptoms, towards alcohol	Finegersh *et al*., 2015 [147]
Alcohol consumption	TET1 expression substantially reduced in rats fed with ethanol	Inhibition of TET1 promoted apoptotic gene expression leading to increased hepatocyte apoptosis	Ji *et al*., 2019 [148]
Air pollution	TET enzymes were upregulated in human PBMCs following 4 hrs of exposure to diluted diesel exhaust	The expression of TET enzymes correlated with proinflammatory cytokine secretion in plasma	Li *et al*., 2022 [149]
Air pollution	Exposure of human bronchial epithelial cells to traffic-related air pollution (TRAP) resulted in lower TET1 expression at 4 hrs but increased after 24 hrs.	Air pollution exposure resulted in time-dependent modulation of TET1 expression which in turn affects 5-hmC levels TRAP regulates TET1 which in turn, modulates 5-hmC levels and resulted in transcriptional activation of downstream genes such as *VEGFA,* known to be associated with lung function	Somineni *et al*., 2016 [150]

HDAC2, histone Deacetylase 2; SAM, S-adenosylmethionine.

## References

[1] Joseph P, Leong D, McKee M, Anand SS, Schwalm JD, Teo K (2017). Reducing the Global Burden of Cardiovascular Disease, Part 1: The Epidemiology and Risk Factors. Circulation Research.

[2] Tada H, Fujino N, Hayashi K, Kawashiri MA, Takamura M (2022). Human genetics and its impact on cardiovascular disease. Journal of Cardiology.

[3] Hajar R (2020). Genetics in Cardiovascular Disease. Heart Views.

[4] Mozaffarian D, Wilson PWF, Kannel WB (2008). Beyond established and novel risk factors: lifestyle risk factors for cardiovascular disease. Circulation.

[5] Landrigan PJ, Fuller R, Acosta NJR, Adeyi O, Arnold R, Basu NN (2018). The Lancet Commission on pollution and health. Lancet.

[6] Cosselman KE, Navas-Acien A, Kaufman JD (2015). Environmental factors in cardiovascular disease. Nature Reviews Cardiology.

[7] Björkegren JLM, Lusis AJ (2022). Atherosclerosis: Recent developments. Cell.

[8] Davignon J, Ganz P (2004). Role of endothelial dysfunction in atherosclerosis. Circulation.

[9] Benincasa G, Coscioni E, Napoli C (2022). Cardiovascular risk factors and molecular routes underlying endothelial dysfunction: Novel opportunities for primary prevention. Biochemical Pharmacology.

[10] Infante T, Costa D, Napoli C (2021). Novel Insights Regarding Nitric Oxide and Cardiovascular Diseases. Angiology.

[11] Korkmaz H, Onalan O (2008). Evaluation of endothelial dysfunction: flow-mediated dilation. Endothelium.

[12] Münzel T, Daiber A (2018). Environmental Stressors and Their Impact on Health and Disease with Focus on Oxidative Stress. Antioxidants & Redox Signaling.

[13] Sandoo A, van Zanten JJCSV, Metsios GS, Carroll D, Kitas GD (2010). The endothelium and its role in regulating vascular tone. The Open Cardiovascular Medicine Journal.

[14] Pi X, Xie L, Patterson C (2018). Emerging Roles of Vascular Endothelium in Metabolic Homeostasis. Circulation Research.

[15] Pugsley MK, Tabrizchi R (2000). The vascular system. An overview of structure and function. Journal of Pharmacological and Toxicological Methods.

[16] Yeboah J, Folsom AR, Burke GL, Johnson C, Polak JF, Post W (2009). Predictive value of brachial flow-mediated dilation for incident cardiovascular events in a population-based study: the multi-ethnic study of atherosclerosis. Circulation.

[17] Anderson TJ, Charbonneau F, Title LM, Buithieu J, Rose MS, Conradson H (2011). Microvascular function predicts cardiovascular events in primary prevention: long-term results from the Firefighters and Their Endothelium (FATE) study. Circulation.

[18] Lind L, Berglund L, Larsson A, Sundström J (2011). Endothelial function in resistance and conduit arteries and 5-year risk of cardiovascular disease. Circulation.

[19] Aboyans V, Lacroix P, Criqui MH (2007). Large and small vessels atherosclerosis: similarities and differences. Progress in Cardiovascular Diseases.

[20] Komarova YA, Kruse K, Mehta D, Malik AB (2017). Protein Interactions at Endothelial Junctions and Signaling Mechanisms Regulating Endothelial Permeability. Circulation Research.

[21] Hu YJ, Wang YD, Tan FQ, Yang WX (2013). Regulation of paracellular permeability: factors and mechanisms. Molecular Biology Reports.

[22] Fung KYY, Fairn GD, Lee WL (2018). Transcellular vesicular transport in epithelial and endothelial cells: Challenges and opportunities. Traffic.

[23] Sumpio BE, Riley JT, Dardik A (2002). Cells in focus: endothelial cell. The International Journal of Biochemistry & Cell Biology.

[24] Ley K, Laudanna C, Cybulsky MI, Nourshargh S (2007). Getting to the site of inflammation: the leukocyte adhesion cascade updated. Nature Reviews Immunology.

[25] Ivetic A, Hoskins Green HL, Hart SJ (2019). L-selectin: A Major Regulator of Leukocyte Adhesion, Migration and Signaling. Frontiers in Immunology.

[26] Wittchen ES (2009). Endothelial signaling in paracellular and transcellular leukocyte transmigration. Frontiers in Bioscience (Landmark Edition).

[27] Peng Z, Shu B, Zhang Y, Wang M (2019). Endothelial Response to Pathophysiological Stress. Arteriosclerosis, Thrombosis, and Vascular Biology.

[28] Qian J, Fulton D (2013). Post-translational regulation of endothelial nitric oxide synthase in vascular endothelium. Frontiers in Physiology.

[29] Fish JE, Yan MS, Matouk CC, St Bernard R, Ho JJD, Gavryushova A (2010). Hypoxic repression of endothelial nitric-oxide synthase transcription is coupled with eviction of promoter histones. The Journal of Biological Chemistry.

[30] Bayaraa O, Inman CK, Thomas SA, Al Jallaf F, Alshaikh M, Idaghdour Y (2022). Hyperglycemic conditions induce rapid cell dysfunction-promoting transcriptional alterations in human aortic endothelial cells. Scientific Reports.

[31] Tamargo IA, Baek KI, Kim Y, Park C, Jo H (2023). Flow-induced re-programming of endothelial cells in atherosclerosis. Nature Reviews Cardiology.

[32] Semenza GL (2007). Hypoxia-inducible factor 1 (HIF-1) pathway. Science’s STKE: Signal Transduction Knowledge Environment.

[33] Majmundar AJ, Wong WJ, Simon MC (2010). Hypoxia-inducible factors and the response to hypoxic stress. Molecular Cell.

[34] Ye JX, Wang SS, Ge M, Wang DJ (2016). Suppression of endothelial PGC-1α is associated with hypoxia-induced endothelial dysfunction and provides a new therapeutic target in pulmonary arterial hypertension. American Journal of Physiology Lung Cellular and Molecular Physiology.

[35] Shoag J, Arany Z (2010). Regulation of hypoxia-inducible genes by PGC-1 alpha. Arteriosclerosis, Thrombosis, and Vascular Biology.

[36] Song W, Zhang CL, Gou L, He L, Gong YY, Qu D (2019). Endothelial TFEB (Transcription Factor EB) Restrains IKK (IκB Kinase)-p65 Pathway to Attenuate Vascular Inflammation in Diabetic db/db Mice. Arteriosclerosis, Thrombosis, and Vascular Biology.

[37] Zhang Y, Zeng C (2016). Role of DNA methylation in cardiovascular diseases. Clinical and Experimental Hypertension.

[38] Xu W, Wang F, Yu Z, Xin F (2016). Epigenetics and Cellular Metabolism. Genetics & Epigenetics.

[39] McGinty RK, Tan S (2015). Nucleosome structure and function. Chemical Reviews.

[40] Wang Z, Chivu AG, Choate LA, Rice EJ, Miller DC, Chu T (2022). Prediction of histone post-translational modification patterns based on nascent transcription data. Nature Genetics.

[41] Sengupta B, Huynh M, Smith CB, McGinty RK, Krajewski W, Lee TH (2022). The Effects of Histone H2B Ubiquitylations on the Nucleosome Structure and Internucleosomal Interactions. Biochemistry.

[42] Shen Y, Wei W, Zhou DX (2015). Histone Acetylation Enzymes Coordinate Metabolism and Gene Expression. Trends in Plant Science.

[43] Chuang JC, Jones PA (2007). Epigenetics and microRNAs. Pediatric Research.

[44] Bernstein BE, Meissner A, Lander ES (2007). The mammalian epigenome. Cell.

[45] Ramazi S, Allahverdi A, Zahiri J (2020). Evaluation of post-translational modifications in histone proteins: A review on histone modification defects in developmental and neurological disorders. Journal of Biosciences.

[46] Singal R, Ginder GD (1999). DNA methylation. Blood.

[47] Moore LD, Fan G (2013). DNA methylation and its basic function. Neuropsychopharmacology.

[48] Rausch C, Hastert FD, Cardoso MC (2020). DNA Modification Readers and Writers and Their Interplay. Journal of Molecular Biology.

[49] Lim DH, Maher ER (2010). DNA methylation: a form of epigenetic control of gene expression. The Obstetrician & Gynaecologist.

[50] Robertson KD (2005). DNA methylation and human disease. Nature Reviews Genetics.

[51] Clouaire T, Stancheva I (2008). Methyl-CpG binding proteins: specialized transcriptional repressors or structural components of chromatin?. Cellular and Molecular Life Sciences.

[52] Wade PA (2001). Methyl CpG-binding proteins and transcriptional repression. BioEssays.

[53] Williams K, Christensen J, Pedersen MT, Johansen JV, Cloos PAC, Rappsilber J (2011). TET1 and hydroxymethylcytosine in transcription and DNA methylation fidelity. Nature.

[54] WYATT GR, COHEN SS (1952). A new pyrimidine base from bacteriophage nucleic acids. Nature.

[55] Pastor WA, Aravind L, Rao A (2013). TETonic shift: biological roles of TET proteins in DNA demethylation and transcription. Nature Reviews Molecular Cell Biology.

[56] Kohli RM, Zhang Y (2013). TET enzymes, TDG and the dynamics of DNA demethylation. Nature.

[57] Koivunen P, Laukka T (2018). The TET enzymes. Cellular and Molecular Life Sciences.

[58] Joshi K, Liu S, Breslin SJP, Zhang J (2022). Mechanisms that regulate the activities of TET proteins. Cellular and Molecular Life Sciences.

[59] Zhang X, Zhang Y, Wang C, Wang X (2023). TET (Ten-eleven translocation) family proteins: structure, biological functions and applications. Signal Transduction and Targeted Therapy.

[60] Liu R, Jin Y, Tang WH, Qin L, Zhang X, Tellides G (2013). Ten-eleven translocation-2 (TET2) is a master regulator of smooth muscle cell plasticity. Circulation.

[61] Peng J, Yang Q, Li AF, Li RQ, Wang Z, Liu LS (2016). Tet methylcytosine dioxygenase 2 inhibits atherosclerosis via upregulation of autophagy in ApoE-/-mice. Oncotarget.

[62] Zhu T, Brown AP, Ji H (2020). The Emerging Role of Ten-Eleven Translocation 1 in Epigenetic Responses to Environmental Exposures. Epigenetics Insights.

[63] Kinney SRM, Pradhan S (2012). Ten eleven translocation enzymes and 5-hydroxymethylation in mammalian development and cancer. Epigenetic Alterations in Oncogenesis.

[64] Ko M, An J, Bandukwala HS, Chavez L, Aijö T, Pastor WA (2013). Modulation of TET2 expression and 5-methylcytosine oxidation by the CXXC domain protein IDAX. Nature.

[65] Dai Y, Chen D, Xu T (2022). DNA Methylation Aberrant in Atherosclerosis. Frontiers in Pharmacology.

[66] Jin Z, Liu Y (2018). DNA methylation in human diseases. Genes & Diseases.

[67] Xia Y, Brewer A, Bell JT (2021). DNA methylation signatures of incident coronary heart disease: findings from epigenome-wide association studies. Clinical Epigenetics.

[68] Flanagan JM (2015). Epigenome-wide association studies (EWAS): past, present, and future. Cancer Epigenetics: Risk Assessment, Diagnosis, Treatment, and Prognosis.

[69] Fernández-Sanlés A, Sayols-Baixeras S, Subirana I, Degano IR, Elosua R (2017). Association between DNA methylation and coronary heart disease or other atherosclerotic events: A systematic review. Atherosclerosis.

[70] Wong LM, Phoon LQ, Wei LK (2021). Epigenetics Modifications in Large-Artery Atherosclerosis: A Systematic Review. Journal of Stroke and Cerebrovascular Diseases.

[71] Lu Q, Schnitzler GR, Vallaster CS, Ueda K, Erdkamp S, Briggs CE (2017). Unliganded estrogen receptor alpha regulates vascular cell function and gene expression. Molecular and Cellular Endocrinology.

[72] Rosenson RS, Brewer HB, Ansell BJ, Barter P, Chapman MJ, Heinecke JW (2016). Dysfunctional HDL and atherosclerotic cardiovascular disease. Nature Reviews Cardiology.

[73] Anderson DR, Poterucha JT, Mikuls TR, Duryee MJ, Garvin RP, Klassen LW (2013). IL-6 and its receptors in coronary artery disease and acute myocardial infarction. Cytokine.

[74] Krolevets M, Cate VT, Prochaska JH, Schulz A, Rapp S, Tenzer S (2023). DNA methylation and cardiovascular disease in humans: a systematic review and database of known CpG methylation sites. Clinical Epigenetics.

[75] Yin Y, Xie Z, Chen D, Guo H, Han M, Zhu Z (2022). Integrated investigation of DNA methylation, gene expression and immune cell population revealed immune cell infiltration associated with atherosclerotic plaque formation. BMC Medical Genomics.

[76] Janjusevic M, Fluca AL, Gagno G, Pierri A, Padoan L, Sorrentino A (2022). Old and Novel Therapeutic Approaches in the Management of Hyperglycemia, an Important Risk Factor for Atherosclerosis. International Journal of Molecular Sciences.

[77] van Rooy MJ, Pretorius E (2014). Obesity, hypertension and hypercholesterolemia as risk factors for atherosclerosis leading to ischemic events. Current Medicinal Chemistry.

[78] Bornfeldt KE, Tabas I (2011). Insulin resistance, hyperglycemia, and atherosclerosis. Cell Metabolism.

[79] Temple ME, Luzier AB, Kazierad DJ (2000). Homocysteine as a risk factor for atherosclerosis. The Annals of Pharmacotherapy.

[80] Kim SK, Kim HJ, Ahn CW, Park SW, Cho YW, Lim SK (2008). Hyperleptinemia as a robust risk factor of coronary artery disease and metabolic syndrome in type 2 diabetic patients. Endocrine Journal.

[81] Rhee M, Lee J, Lee EY, Yoon KH, Lee SH (2024). Lipid Variability Induces Endothelial Dysfunction by Increasing Inflammation and Oxidative Stress. Endocrinology and Metabolism.

[82] Lai WKC, Kan MY (2015). Homocysteine-Induced Endothelial Dysfunction. Annals of Nutrition & Metabolism.

[83] Liu B, Qiao J, Hu J, Fan M, Zhao Y, Su H (2020). Leptin promotes endothelial dysfunction in chronic kidney disease by modulating the MTA1-mediated WNT/β-catenin pathway. Molecular and Cellular Biochemistry.

[84] Janaszak-Jasiecka A, Siekierzycka A, Płoska A, Dobrucki IT, Kalinowski L (2021). Endothelial Dysfunction Driven by Hypoxia-The Influence of Oxygen Deficiency on NO Bioavailability. Biomolecules.

[85] Higashi Y, Maruhashi T, Noma K, Kihara Y (2014). Oxidative stress and endothelial dysfunction: clinical evidence and therapeutic implications. Trends in Cardiovascular Medicine.

[86] Giebe S, Cockcroft N, Hewitt K, Brux M, Hofmann A, Morawietz H (2017). Cigarette smoke extract counteracts atheroprotective effects of high laminar flow on endothelial function. Redox Biology.

[87] Chien S (2008). Effects of disturbed flow on endothelial cells. Annals of Biomedical Engineering.

[88] Pepin ME, Schiano C, Miceli M, Benincasa G, Mansueto G, Grimaldi V (2021). The human aortic endothelium undergoes dose-dependent DNA methylation in response to transient hyperglycemia. Experimental Cell Research.

[89] Dunn J, Qiu H, Kim S, Jjingo D, Hoffman R, Kim CW (2014). Flow-dependent epigenetic DNA methylation regulates endothelial gene expression and atherosclerosis. The Journal of Clinical Investigation.

[90] Jia SJ, Lai YQ, Zhao M, Gong T, Zhang BK (2013). Homocysteine-induced hypermethylation of DDAH2 promoter contributes to apoptosis of endothelial cells. Die Pharmazie.

[91] Mudersbach T, Siuda D, Kohlstedt K, Fleming I (2019). Epigenetic control of the angiotensin-converting enzyme in endothelial cells during inflammation. PLoS ONE.

[92] Vigorelli V, Resta J, Bianchessi V, Lauri A, Bassetti B, Agrifoglio M (2019). Abnormal DNA Methylation Induced by Hyperglycemia Reduces CXCR 4 Gene Expression in CD 34^+^ Stem Cells. Journal of the American Heart Association.

[93] Binjawhar DN, Alhazmi AT, Bin Jawhar WN, MohammedSaeed W, Safi SZ (2023). Hyperglycemia-induced oxidative stress and epigenetic regulation of ET-1 gene in endothelial cells. Frontiers in Genetics.

[94] Paneni F, Mocharla P, Akhmedov A, Costantino S, Osto E, Volpe M (2012). Gene silencing of the mitochondrial adaptor p66(Shc) suppresses vascular hyperglycemic memory in diabetes. Circulation Research.

[95] Aref-Eshghi E, Biswas S, Chen C, Sadikovic B, Chakrabarti S (2020). Glucose-induced, duration-dependent genome-wide DNA methylation changes in human endothelial cells. American Journal of Physiology Cell Physiology.

[96] Scisciola L, Rizzo MR, Marfella R, Cataldo V, Fontanella RA, Boccalone E (2021). New insight in molecular mechanisms regulating SIRT6 expression in diabetes: Hyperglycaemia effects on SIRT6 DNA methylation. Journal of Cellular Physiology.

[97] Wang X, Li Q, He S, Bai J, Ma C, Zhang L (2022). LncRNA FENDRR with m6A RNA methylation regulates hypoxia-induced pulmonary artery endothelial cell pyroptosis by mediating DRP1 DNA methylation. Molecular Medicine.

[98] Xu X, Tan X, Hulshoff MS, Wilhelmi T, Zeisberg M, Zeisberg EM (2016). Hypoxia-induced endothelial-mesenchymal transition is associated with RASAL1 promoter hypermethylation in human coronary endothelial cells. FEBS Letters.

[99] Peñaloza E, Soto-Carrasco G, Krause BJ (2020). MiR-21-5p directly contributes to regulating eNOS expression in human artery endothelial cells under normoxia and hypoxia. Biochemical Pharmacology.

[100] Jiang YZ, Jiménez JM, Ou K, McCormick ME, Zhang LD, Davies PF (2014). Hemodynamic disturbed flow induces differential DNA methylation of endothelial Kruppel-Like Factor 4 promoter in vitro and in vivo. Circulation Research.

[101] Zhou J, Li YS, Wang KC, Chien S (2014). Epigenetic Mechanism in Regulation of Endothelial Function by Disturbed Flow: Induction of DNA Hypermethylation by DNMT1. Cellular and Molecular Bioengineering.

[102] Ma SC, Hao YJ, Jiao Y, Wang YH, Xu LB, Mao CY (2017). Homocysteine induced oxidative stress through TLR4/NF κB/DNMT1 mediated LOX 1 DNA methylation in endothelial cells. Molecular Medicine Reports.

[103] Kim CS, Kim YR, Naqvi A, Kumar S, Hoffman TA, Jung SB (2011). Homocysteine promotes human endothelial cell dysfunction via site-specific epigenetic regulation of p66shc. Cardiovascular Research.

[104] Jamaluddin MDS, Chen I, Yang F, Jiang X, Jan M, Liu X (2007). Homocysteine inhibits endothelial cell growth via DNA hypomethylation of the cyclin A gene. Blood.

[105] Wang L, Mao B, Fan K, Sun R, Zhang J, Liang H (2022). ROS attenuates TET2-dependent ZO-1 epigenetic expression in cerebral vascular endothelial cells. Fluids and Barriers of the CNS.

[106] Rhead B, Shao X, Quach H, Ghai P, Barcellos LF, Bowcock AM (2020). Global expression and CpG methylation analysis of primary endothelial cells before and after TNFa stimulation reveals gene modules enriched in inflammatory and infectious diseases and associated DMRs. PLoS ONE.

[107] Yan Z, Deng Y, Jiao F, Guo J, Ou H (2017). Lipopolysaccharide Downregulates Kruppel-Like Factor 2 (KLF2) via Inducing DNMT1-Mediated Hypermethylation in Endothelial Cells. Inflammation.

[108] Mitra S, Khaidakov M, Lu J, Ayyadevara S, Szwedo J, Wang XW (2011). Prior exposure to oxidized low-density lipoprotein limits apoptosis in subsequent generations of endothelial cells by altering promoter methylation. American Journal of Physiology Heart and Circulatory Physiology.

[109] Zhaolin Z, Jiaojiao C, Peng W, Yami L, Tingting Z, Jun T (2019). OxLDL induces vascular endothelial cell pyroptosis through miR-125a-5p/TET2 pathway. Journal of Cellular Physiology.

[110] Kumar A, Kumar S, Vikram A, Hoffman TA, Naqvi A, Lewarchik CM (2013). Histone and DNA methylation-mediated epigenetic downregulation of endothelial Kruppel-like factor 2 by low-density lipoprotein cholesterol. Arteriosclerosis, Thrombosis, and Vascular Biology.

[111] Kim YR, Kim CS, Naqvi A, Kumar A, Kumar S, Hoffman TA (2012). Epigenetic upregulation of p66shc mediates low-density lipoprotein cholesterol-induced endothelial cell dysfunction. American Journal of Physiology Heart and Circulatory Physiology.

[112] Zeng H, Kong X, Zhang H, Chen Y, Cai S, Luo H (2020). Inhibiting DNA methylation alleviates cigarette smoke extract-induced dysregulation of Bcl-2 and endothelial apoptosis. Tobacco Induced Diseases.

[113] Yang M, Chen P, Peng H, Zhang H, Chen Y, Cai S (2015). Cigarette smoke extract induces aberrant cytochrome-c oxidase subunit II methylation and apoptosis in human umbilical vascular endothelial cells. American Journal of Physiology Cell Physiology.

[114] Peng H, Guo T, Chen Z, Zhang H, Cai S, Yang M (2019). Hypermethylation of mitochondrial transcription factor A induced by cigarette smoke is associated with chronic obstructive pulmonary disease. Experimental Lung Research.

[115] Pautz A, Li H, Kleinert H (2021). Regulation of NOS expression in vascular diseases. Frontiers in Bioscience (Landmark Edition).

[116] Krause BJ, Costello PM, Muñoz-Urrutia E, Lillycrop KA, Hanson MA, Casanello P (2013). Role of DNA methyltransferase 1 on the altered eNOS expression in human umbilical endothelium from intrauterine growth restricted fetuses. Epigenetics.

[117] Steensma DP (2018). Clinical consequences of clonal hematopoiesis of indeterminate potential. Hematology.

[118] Ganguly BB, Banerjee D, Agarwal MB (2018). Impact of chromosome alterations, genetic mutations and clonal hematopoiesis of indeterminate potential (CHIP) on the classification and risk stratification of MDS. Blood Cells, Molecules & Diseases.

[119] Fuster JJ, MacLauchlan S, Zuriaga MA, Polackal MN, Ostriker AC, Chakraborty R (2017). Clonal hematopoiesis associated with TET2 deficiency accelerates atherosclerosis development in mice. Science.

[120] Jaiswal S, Fontanillas P, Flannick J, Manning A, Grauman PV, Mar BG (2014). Age-related clonal hematopoiesis associated with adverse outcomes. The New England Journal of Medicine.

[121] Kwon YM, Park JH, Kim H, Shim YM, Kim J, Han J (2007). Different susceptibility of increased DNMT1 expression by exposure to tobacco smoke according to histology in primary non-small cell lung cancer. Journal of Cancer Research and Clinical Oncology.

[122] Bönsch D, Lenz B, Fiszer R, Frieling H, Kornhuber J, Bleich S (2006). Lowered DNA methyltransferase (DNMT-3b) mRNA expression is associated with genomic DNA hypermethylation in patients with chronic alcoholism. Journal of Neural Transmission.

[123] Sakharkar AJ, Tang L, Zhang H, Chen Y, Grayson DR, Pandey SC (2014). Effects of acute ethanol exposure on anxiety measures and epigenetic modifiers in the extended amygdala of adolescent rats. The International Journal of Neuropsychopharmacology.

[124] Sultan S, AlMalki S (2023). Analysis of global DNA methylation and epigenetic modifiers (DNMTs and HDACs) in human foetal endothelium exposed to gestational and type 2 diabetes. Epigenetics.

[125] Maugeri A, Mazzone MG, Giuliano F, Vinciguerra M, Basile G, Barchitta M (2018). Curcumin Modulates DNA Methyltransferase Functions in a Cellular Model of Diabetic Retinopathy. Oxidative Medicine and Cellular Longevity.

[126] Liu Y, Tian X, Liu S, Liu D, Li Y, Liu M (2020). DNA hypermethylation: A novel mechanism of CREG gene suppression and atherosclerogenic endothelial dysfunction. Redox Biology.

[127] Liu P, Liu Y, Liu H, Pan X, Li Y, Usa K (2018). Role of DNA De Novo (De)Methylation in the Kidney in Salt-Induced Hypertension. Hypertension.

[128] Nowrasteh G, Zand A, Raposa LB, Szabó L, Tomesz A, Molnár R (2023). Fruit Extract, Rich in Polyphenols and Flavonoids, Modifies the Expression of *DNMT* and *HDAC* Genes Involved in Epigenetic Processes. Nutrients.

[129] Hunter DJ, James L, Hussey B, Wadley AJ, Lindley MR, Mastana SS (2019). Impact of aerobic exercise and fatty acid supplementation on global and gene-specific DNA methylation. Epigenetics.

[130] Dunn J, Simmons R, Thabet S, Jo H (2015). The role of epigenetics in the endothelial cell shear stress response and atherosclerosis. The International Journal of Biochemistry & Cell Biology.

[131] De Cabo SF, Santos J, Fernández-Piqueras J (1995). Molecular and cytological evidence of S-adenosyl-L-homocysteine as an innocuous undermethylating agent in vivo. Cytogenetics and Cell Genetics.

[132] Zhang H, Liu Z, Ma S, Zhang H, Kong F, He Y (2016). Ratio of S-adenosylmethionine to S-adenosylhomocysteine as a sensitive indicator of atherosclerosis. Molecular Medicine Reports.

[133] Niemann B, Rohrbach S, Miller MR, Newby DE, Fuster V, Kovacic JC (2017). Oxidative Stress and Cardiovascular Risk: Obesity, Diabetes, Smoking, and Pollution: Part 3 of a 3-Part Series. Journal of the American College of Cardiology.

[134] van der Wijst MGP, Venkiteswaran M, Chen H, Xu GL, Plösch T, Rots MG (2015). Local chromatin microenvironment determines DNMT activity: from DNA methyltransferase to DNA demethylase or DNA dehydroxymethylase. Epigenetics.

[135] Hepburn PA, Margison GP, Tisdale MJ (1991). Enzymatic methylation of cytosine in DNA is prevented by adjacent O6-methylguanine residues. The Journal of Biological Chemistry.

[136] O’Hagan HM, Wang W, Sen S, Destefano Shields C, Lee SS, Zhang YW (2011). Oxidative damage targets complexes containing DNA methyltransferases, SIRT1, and polycomb members to promoter CpG Islands. Cancer Cell.

[137] Ciccarone F, Castelli S, Ioannilli L, Ciriolo MR (2019). High Dietary Fat Intake Affects DNA Methylation/Hydroxymethylation in Mouse Heart: Epigenetic Hints for Obesity-Related Cardiac Dysfunction. Molecular Nutrition & Food Research.

[138] Pei S, Liu R, Ma Q, Jiang P, He X, Qi Z (2024). Punicalagin prevents obesity-related cardiac dysfunction through promoting DNA demethylation in mice. Food Science and Human Wellness.

[139] Peng J, Tang ZH, Ren Z, He B, Zeng Y, Liu LS (2017). TET2 Protects against oxLDL-Induced HUVEC Dysfunction by Upregulating the CSE/H_2_S System. Frontiers in Pharmacology.

[140] Li A, Tan L, Zhang S, Tao J, Wang Z, Wei D (2021). Low shear stress-induced endothelial mesenchymal transformation via the down-regulation of TET2. Biochemical and Biophysical Research Communications.

[141] Yang Q, Li X, Li R, Peng J, Wang Z, Jiang Z (2016). Low Shear Stress Inhibited Endothelial Cell Autophagy Through TET2 Downregulation. Annals of Biomedical Engineering.

[142] Chen J, Zhang J, Wu J, Zhang S, Liang Y, Zhou B (2021). Low shear stress induced vascular endothelial cell pyroptosis by TET2/SDHB/ROS pathway. Free Radical Biology & Medicine.

[143] Kaur G, Batra S (2020). Regulation of DNA methylation signatures on NF-κB and STAT3 pathway genes and TET activity in cigarette smoke extract-challenged cells/COPD exacerbation model in vitro. Cell Biology and Toxicology.

[144] Wain LV, Shrine N, Miller S, Jackson VE, Ntalla I, Soler Artigas M (2015). Novel insights into the genetics of smoking behaviour, lung function, and chronic obstructive pulmonary disease (UK BiLEVE): a genetic association study in UK Biobank. The Lancet Respiratory Medicine.

[145] Jessop P, Toledo-Rodriguez M (2018). Hippocampal *TET1* and *TET2* Expression and DNA Hydroxymethylation Are Affected by Physical Exercise in Aged Mice. Frontiers in Cell and Developmental Biology.

[146] Guidotti A, Dong E, Gavin DP, Veldic M, Zhao W, Bhaumik DK (2013). DNA methylation/demethylation network expression in psychotic patients with a history of alcohol abuse. Alcoholism, Clinical and Experimental Research.

[147] Finegersh A, Ferguson C, Maxwell S, Mazariegos D, Farrell D, Homanics GE (2015). Repeated vapor ethanol exposure induces transient histone modifications in the brain that are modified by genotype and brain region. Frontiers in Molecular Neuroscience.

[148] Ji C, Nagaoka K, Zou J, Casulli S, Lu S, Cao KY (2019). Chronic ethanol-mediated hepatocyte apoptosis links to decreased TET1 and 5-hydroxymethylcytosine formation. FASEB Journal.

[149] Li H, Ryu MH, Orach J, Yuen A, Lau KSK, Yeung C (2022). Acute air pollution exposure increases TET enzymes in human PBMCs. The Journal of Allergy and Clinical Immunology.

[150] Somineni HK, Zhang X, Biagini Myers JM, Kovacic MB, Ulm A, Jurcak N (2016). Ten-eleven translocation 1 (TET1) methylation is associated with childhood asthma and traffic-related air pollution. The Journal of Allergy and Clinical Immunology.

[151] Xiao M, Yang H, Xu W, Ma S, Lin H, Zhu H (2012). Inhibition of α-KG-dependent histone and DNA demethylases by fumarate and succinate that are accumulated in mutations of FH and SDH tumor suppressors. Genes & Development.

[152] Xu W, Yang H, Liu Y, Yang Y, Wang P, Kim SH (2011). Oncometabolite 2-hydroxyglutarate is a competitive inhibitor of α-ketoglutarate-dependent dioxygenases. Cancer Cell.

[153] Kaplánek R, Kejík Z, Hajduch J, Veselá K, Kučnirová K, Skaličková M (2023). TET protein inhibitors: Potential and limitations. Biomedicine & Pharmacotherapy.

[154] Burr S, Caldwell A, Chong M, Beretta M, Metcalf S, Hancock M (2018). Oxygen gradients can determine epigenetic asymmetry and cellular differentiation via differential regulation of Tet activity in embryonic stem cells. Nucleic Acids Research.

[155] Sciacovelli M, Gonçalves E, Johnson TI, Zecchini VR, da Costa ASH, Gaude E (2016). Fumarate is an epigenetic modifier that elicits epithelial-to-mesenchymal transition. Nature.

[156] Delatte B, Jeschke J, Defrance M, Bachman M, Creppe C, Calonne E (2015). Genome-wide hydroxymethylcytosine pattern changes in response to oxidative stress. Scientific Reports.

[157] Green HLH, Brewer AC (2020). Dysregulation of 2-oxoglutarate-dependent dioxygenases by hyperglycaemia: does this link diabetes and vascular disease?. Clinical Epigenetics.

[158] Moselhy SS, Demerdash SH (2003). Plasma homocysteine and oxidative stress in cardiovascular disease. Disease Markers.

[159] Faraci FM, Lentz SR (2004). Hyperhomocysteinemia, oxidative stress, and cerebral vascular dysfunction. Stroke.

[160] Yin R, Mao SQ, Zhao B, Chong Z, Yang Y, Zhao C (2013). Ascorbic acid enhances Tet-mediated 5-methylcytosine oxidation and promotes DNA demethylation in mammals. Journal of the American Chemical Society.

[161] Lykkesfeldt J, Loft S, Nielsen JB, Poulsen HE (1997). Ascorbic acid and dehydroascorbic acid as biomarkers of oxidative stress caused by smoking. The American Journal of Clinical Nutrition.

[162] Yang H, Lin H, Xu H, Zhang L, Cheng L, Wen B (2014). TET-catalyzed 5-methylcytosine hydroxylation is dynamically regulated by metabolites. Cell Research.

[163] Yuan EF, Yang Y, Cheng L, Deng X, Chen SM, Zhou X (2019). Hyperglycemia affects global 5-methylcytosine and 5-hydroxymethylcytosine in blood genomic DNA through upregulation of SIRT6 and TETs. Clinical Epigenetics.

[164] Wu D, Hu D, Chen H, Shi G, Fetahu IS, Wu F (2018). Glucose-regulated phosphorylation of TET2 by AMPK reveals a pathway linking diabetes to cancer. Nature.

[165] Besaratinia A, Caceres A, Tommasi S (2022). DNA Hydroxymethylation in Smoking-Associated Cancers. International Journal of Molecular Sciences.

[166] Dong C, Chen J, Zheng J, Liang Y, Yu T, Liu Y (2020). 5-Hydroxymethylcytosine signatures in circulating cell-free DNA as diagnostic and predictive biomarkers for coronary artery disease. Clinical Epigenetics.

[167] Arvinden VR, Deva Magendhra Rao AK, Rajkumar T, Mani S (2017). Regulation and functional significance of 5-hydroxymethylcytosine in cancer. Epigenomes.

[168] Shirwany NA, Zou MH (2014). AMPK: a cellular metabolic and redox sensor. A minireview. Frontiers in Bioscience (Landmark Edition).

[169] Wang Y, Hu J, Wu S, Fleishman JS, Li Y, Xu Y (2023). Targeting epigenetic and posttranslational modifications regulating ferroptosis for the treatment of diseases. Signal Transduction and Targeted Therapy.

[170] Bauer C, Göbel K, Nagaraj N, Colantuoni C, Wang M, Müller U (2015). Phosphorylation of TET proteins is regulated via O-GlcNAcylation by the O-linked N-acetylglucosamine transferase (OGT). The Journal of Biological Chemistry.

[171] Slawson C, Copeland RJ, Hart GW (2010). O-GlcNAc signaling: a metabolic link between diabetes and cancer?. Trends in Biochemical Sciences.

[172] Chen Y, Zhao X, Wu H (2019). Metabolic Stress and Cardiovascular Disease in Diabetes Mellitus: The Role of Protein O-GlcNAc Modification. Arteriosclerosis, Thrombosis, and Vascular Biology.

[173] Lee A, Miller D, Henry R, Paruchuri VDP, O’Meally RN, Boronina T (2016). Combined Antibody/Lectin Enrichment Identifies Extensive Changes in the O-GlcNAc Sub-proteome upon Oxidative Stress. Journal of Proteome Research.

[174] Zhang YW, Wang Z, Xie W, Cai Y, Xia L, Easwaran H (2017). Acetylation Enhances TET2 Function in Protecting against Abnormal DNA Methylation during Oxidative Stress. Molecular Cell.

[175] Barabási AL, Gulbahce N, Loscalzo J (2011). Network medicine: a network-based approach to human disease. Nature Reviews Genetics.

[176] Vinciguerra M (2023). The Potential for Artificial Intelligence Applied to Epigenetics. Mayo Clinic Proceedings: Digital Health.

[177] Wu G, Zhang X, Gao F (2021). The epigenetic landscape of exercise in cardiac health and disease. Journal of Sport and Health Science.

[178] Keshawarz A, Joehanes R, Guan W, Huan T, DeMeo DL, Grove ML (2022). Longitudinal change in blood DNA epigenetic signature after smoking cessation. Epigenetics.

[179] Mulrenan C, Petticrew M, Wallop H (2023). Big Macs and the Beano: Is it time for the comic to drop the junk food brands?. BMJ (Clinical Research Ed).

